# Na^+^/K^+^-ATPase Modulates Purinergic P2X3 Receptor Function to Drive Bone Cancer Pain

**DOI:** 10.34133/research.0932

**Published:** 2025-10-20

**Authors:** Songqiang Huang, Bo Peng, Wanting Dong, Jiapeng He, Hanbin Chen, Jin-Song Bian

**Affiliations:** ^1^ Affiliated Hospital of Hunan University, School of Biomedical Sciences, Hunan University, Changsha 410082, China.; ^2^ School of Medicine, Southern University of Science and Technology, Shenzhen 518055, China.; ^3^ Department of Pharmacology, Joint Laboratory of Guangdong-Hong Kong Universities for Vascular Homeostasis and Diseases, SUSTech Homeostatic Medicine Institute, School of Medicine, Southern University of Science and Technology, Shenzhen 518055, China.

## Abstract

Bone cancer pain (BCP) is one of the most common types of chronic pain in cancer patients, with a prevalence of up to 75%. However, the pathological mechanism and therapeutic approaches are limited. Here, we demonstrated that Na^+^/K^+^-ATPase α1 (NKAα1) is a critical regulator of nociception through interaction with purinergic P2X3 receptor (P2X3R) in the dorsal root ganglion (DRG). Conditional knockout of NKAα1 in transient receptor potential vanilloid 1-positive (TRPV1^+^) neurons led to an increase in P2X3R-dependent Ca^2+^ influx and neuronal hyperexcitability and also promoted pain hypersensitivity in BCP model mice. In addition, NKAα1 knockout in TRPV1^+^ neurons further enhanced C-C motif chemokine ligand 5 release, thereby exacerbating spinal glial cell activation and pain hypersensitivity in BCP mice. DR5-12D, a monoclonal antibody to stabilize the expression of NKAα1, markedly inhibited the hyperexcitability of DRG nociceptors and ameliorated pain hypersensitivity in BCP mice. Overall, NKAα1 modulates P2X3R-dependent Ca^2+^ influx and the excitability of DRG nociceptors, thereby providing valuable theoretical guidance for the treatment of BCP.

## Introduction

Cancer-related pain is highly prevalent and substantially impairs patients’ quality of life [1], and bone metastasis is a common feature of advanced cancers, often resulting in pronounced skeletal degradation and debilitating bone cancer pain (BCP) [2]. BCP is a mixed-pain syndrome, involving both neuropathic and inflammatory mechanisms, and is characterized by allodynia, hyperalgesia, and persistent spontaneous pain [3]. Nearly 46% of cancer patients exhibit BCP, ultimately leading to a marked deterioration in the quality of life among cancer patients [4]. Despite the administration of potent analgesics, including opioids, a substantial proportion of patients with BCP still fail to achieve adequate pain relief [3,5]. Therefore, gaining insight into the pathophysiological pathways of BCP and exploring innovative therapeutic strategies remain essential to improve clinical outcomes.

Dorsal root ganglion (DRG) neuronal injury and tumor-induced primary afferent sensory nerve fiber injury are possible factors contributing to the sustained pain hypersensitivity observed in BCP [6,7]. Located at the initial relay point of the somatosensory pathway, DRG neurons play a pivotal role in conveying peripheral sensory information to the spinal cord and ultimately the brain [8]. In rodent models of BCP, upregulated purinergic P2X3 receptor (P2X3R) signaling in DRG has been implicated in promoting neuronal hyperexcitability and pain hypersensitivity [9,10]. Activation of P2X3R leads to increased intracellular calcium ion (Ca^2+^) levels and action potential in sensory neurons [11,12]. These studies suggest that P2X3R in sensory neurons promotes pain hypersensitivity by mediating Ca^2+^ influx and neuronal excitability. However, the upstream molecular mechanisms underlying P2X3R-mediated pain behavior remain insufficiently explored.

Na^+^/K^+^-ATPase (NKA), an essential membrane pump, exchanges intracellular sodium ions (Na^+^) and extracellular potassium ions (K^+^) in a 3:2 ratio, thereby maintaining the resting membrane potential and supporting neuronal excitability [13,14]. NKA, a membrane-bound enzyme formed by α, β, and γ subunits, relies on the α subunit for its catalytic function, with the α1 isoform abundantly expressed in neurons as well as glia [15,16]. Studies have shown that NKA activity in cardiomyocytes and neurons is closely related to intracellular Ca^2+^ overload and cell damage [17–19]. In addition, NKA activation suppresses excitatory afferent input, thereby modulating the threshold of somatic sensation [15,20]. Our earlier work revealed that the complex of Na^+^/K^+^-ATPase α1 (NKAα1) and purinergic P2X7 receptor (P2X7R) in microglia regulates neuroinflammation and the excitability of hippocampal neurons [21]. However, it remains unclear whether NKAα1 is associated with P2X3R and involved in regulating pain hypersensitivity and whether NKAα1 could serve as a novel target for BCP treatment.

In this study, we demonstrated that NKAα1 expression was markedly reduced in tumor cell implantation (TCI) and partial sciatic nerve ligation (PSNL) models. NKAα1 interacts with P2X3R in DRG neurons and further inhibits P2X3R-dependent Ca^2+^ influx. Loss of NKAα1 aggravates TCI-induced neuronal hyperexcitability and also modulates glial cell activation in the spinal cord via controlling the release of C-C motif chemokine ligand 5 (CCL5) from transient receptor potential vanilloid 1-positive (TRPV1^+^) neurons in DRG. The monoclonal antibody DR5-12D, which specifically targets the DR region (^897^DVEDSYGQQWTYEQR^911^) of NKAα1, markedly reduced TCI-induced pain-like behavior by inhibiting the hyperexcitability of nociceptive neurons.

## Results

### NKAα1 interacts with P2X3R in DRG neurons

**Fig. 1.**
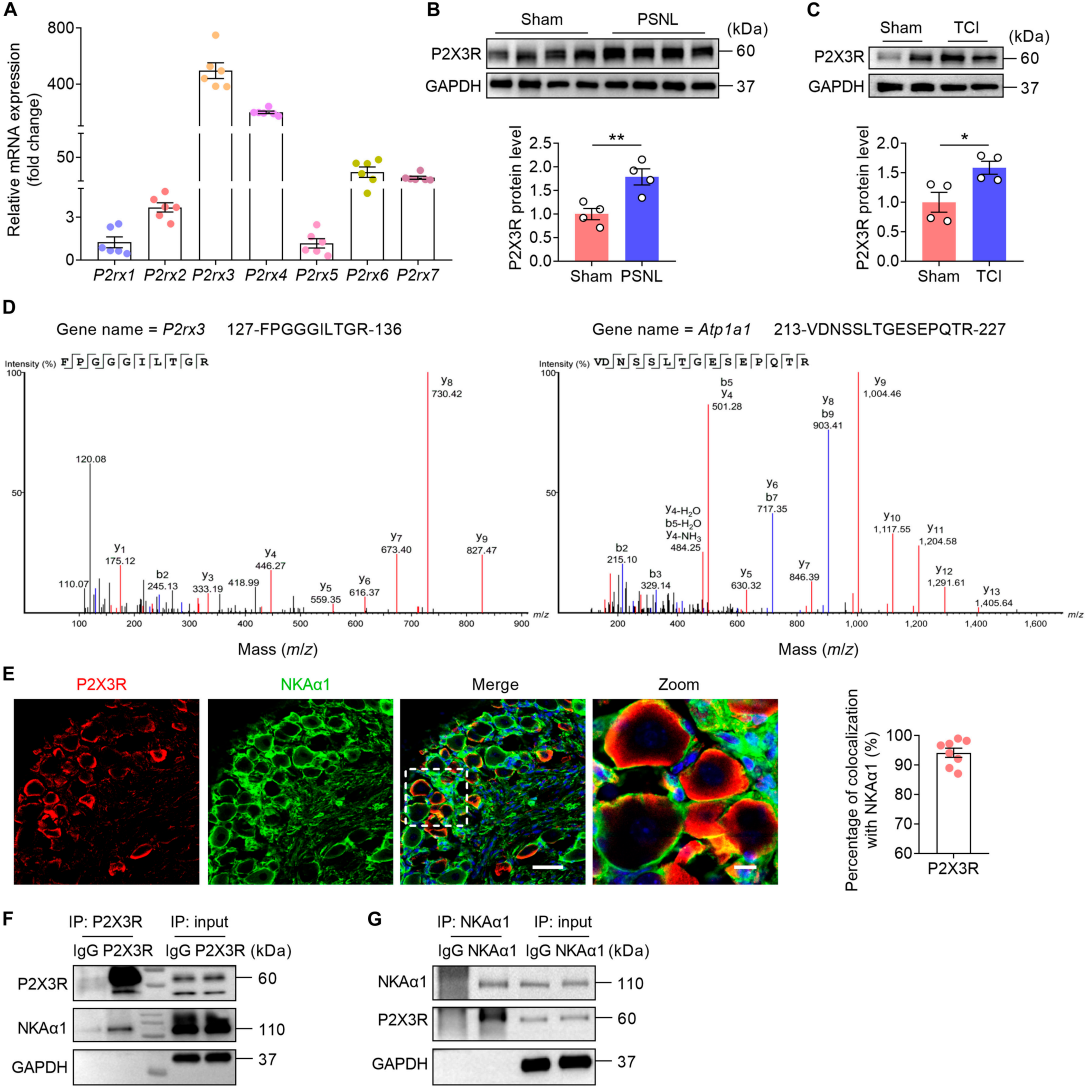
Na^+^/K^+^-ATPase α1 (NKAα1) interacts with P2X3 receptor (P2X3R) in dorsal root ganglion (DRG) neurons. (A) Quantitative polymerase chain reaction (qPCR) analysis showing the messenger RNA (mRNA) expression of *P2rx1*, *P2rx2*, *P2rx3*, *P2rx4*, *P2rx5*, *P2rx6*, and *P2rx7* in the DRG (*n* = 6). (B) Representative western blots and analyses of P2X3R in the DRG of sham and partial sciatic nerve ligation (PSNL) mice (*n* = 4). (C) Representative western blots and analyses of P2X3R in the DRG of sham and tumor cell implantation (TCI) mice (*n* = 4). (D) Annotated tandem mass spectrometry (MS/MS) spectra of 127-FPGGGILTGR-136 from P2X3R and 213-VDNSSLTGESEPQTR-227 from NKAα1 in anti-P2X3R immunoprecipitates from the DRG of 8-week naive mice. (E) Representative images and colocalization analyses of P2X3R (red) and NKAα1 (green) in DRG (*n* = 8). The scale bars in (E) indicate 50 μm (left) and 10 μm (right, Zoom). (F and G) Co-immunoprecipitation analysis showing the interaction between NKAα1 and P2X3R in DRG. Data are presented as mean ± standard error of the mean (SEM). Statistical analysis by unpaired Student *t* test in (B) and (C). *P < 0.05; **P < 0.01. GAPDH, glyceraldehyde-3-phosphate dehydrogenase; IP, immunoprecipitation; IgG, immunoglobulin G.

Mammalian purinergic P2X receptors are divided into 7 subtypes (*P2rx1* to *P2rx7*) [22]. Activation of purinergic P2X receptors occurs upon binding to adenosine 5′-triphosphate (ATP), resulting in the opening of channels permeable to ions, which play a crucial role in the mechanisms underlying cancer pain [23]. Compared to other P2X receptor subtypes, the messenger RNA (mRNA) expression of *P2rx3* was highest in DRG tissues of mice (Fig. 1A). Moreover, the protein expression of P2X3R was increased in DRG from PSNL and TCI models (Fig. 1B and C). P2X3R is a membrane protein that has been shown to interact with G protein-coupled receptor 151 (GPR151) and acid-sensing ion channel 3 (ASIC3), thereby regulating the processes underlying neuropathic and inflammatory pain [24,25]. However, it is not known whether P2X3R also has a similar mechanism in the BCP model. A total of 122 P2X3R-interacting proteins (fold change > 2, Data S1) were found using liquid chromatography–tandem mass spectrometry (LC–MS/MS). Based on confidence score ranking, periaxin was identified as the top protein, playing a crucial role in maintaining myelin sheath stability in peripheral neurons [26]. Plectin ranked second, primarily contributing to the establishment and stabilization of the neuronal microtubule network, thereby supporting neuronal structure and integrity [27]. Notably, the membrane protein NKAα1 emerged as the third most enriched interactor (Fig. 1D); its principal function is to regulate ion gradients across the cell membrane, including Na^+^, K^+^, and Ca^2+^ [28,29]. Given the critical involvement of Ca^2+^ influx in pain transmission and modulation, we selected NKAα1 for further investigation to elucidate its potential role in pain-related signaling pathways. Furthermore, immunofluorescence and co-immunoprecipitation (Co-IP) experiments showed that P2X3R colocalizes with and interacts with NKAα1 in the cell bodies of DRG neurons (Fig. 1E to G). The results suggest that NKAα1 may contribute to the modulation of P2X3R-driven pain-like behavior.

### NKAα1 is widely distributed in all sensory neurons of DRG

**Fig. 2.**
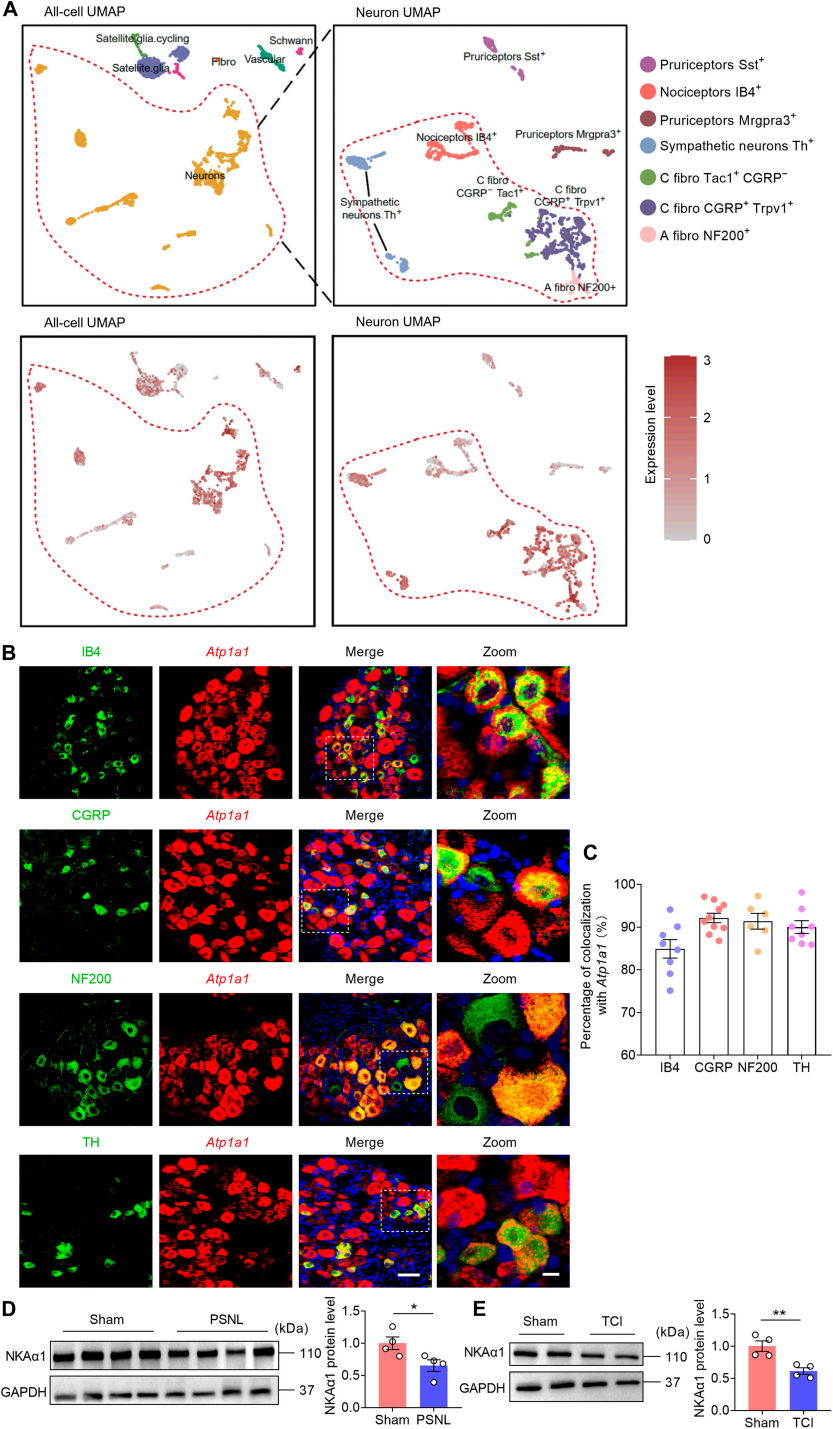
NKAα1 is expressed in all types of neurons in DRG. (A) Uniform manifold approximation and projection (UMAP) plots showing the single-cell RNA sequencing (scRNA-seq) results of NKAα1 distribution in the DRG. Raw data of scRNA-seq from Zeisel et al., *Cell*, 2018 [30]. (B and C) Representative images (B) and colocalization analyses (C) of NKAα1 (*Atp1a1* gene, red), isolectin B4 (IB4; nonpeptidergic C-fiber marker, green), calcitonin gene-related peptide (CGRP; peptidergic C-fiber marker, green), neurofilament-200 (NF200; myelinated A-fiber marker, green), and tyrosine hydroxylase (TH; low-threshold C-fiber marker, green) in DRG (*n* = 8). The white box shows the zoom area. (D) Representative western blots and analyses of NKAα1 in the DRG of sham and PSNL mice (*n* = 4). (E) Representative western blots and analyses of NKAα1 in the DRG of sham and TCI mice (*n* = 4). The scale bars in (B) indicate 50 μm (left) and 10 μm (right, Zoom). Data are presented as mean ± SEM. Statistical analysis by unpaired Student *t* test in (D) and (E). *P < 0.05; **P < 0.01.

To investigate the cellular distribution of NKA subunits, particularly α1, in DRG neurons, we analyzed publicly available single-cell RNA sequencing (scRNA-seq) datasets [30]. *Atp1a1*, the gene encoding NKAα1, was broadly expressed across most cell types, with the exception of fibroblasts (Fig. 2A).

Given that the α subunit serves as the core catalytic component of NKA [21], our analysis concentrated on its isoforms. Among them, α1 exhibited the greatest transcript abundance in neurons and showed relatively uniform expression across various sensory neuron subtypes, including nociceptors (Fig. S1A and B). By contrast, α2 and α3 were predominantly enriched in neuronal populations associated with itch processing (Fig. S1A to D), such as Tac1^+^, Mrgpra3^+^, and Sst^+^ neurons [31–33]. Further, we utilized in situ hybridization and immunofluorescence to observe the distribution characteristics of NKAα1 in the DRG of mice and found that NKAα1 was colocalized with isolectin B4 (IB4; labeling nonpeptidergic C-type fiber), calcitonin gene-related peptide (CGRP; indicative of peptidergic C-type fibers), neurofilament-200 (NF200; marking myelinated A-type fibers), tyrosine hydroxylase (TH; associated with low-threshold C fibers), and TRPV1 (indicative of nociceptive neurons). Furthermore, both the expression abundance and distribution pattern of NKAα1 appeared consistent across various sensory neuron subtypes (Fig. 2B and C and Fig. S1E and F). Our data further revealed that NKAα1 protein expression was markedly reduced in the DRG of mice under PSNL and TCI conditions (Fig. 2D and E). These results suggest that NKAα1 expression within DRG neurons is likely involved in the regulation of pain-related behaviors in mice.

### Conditional knockout of NKAα1 in nociceptive neurons promotes pain hypersensitivity

**Fig. 3.**
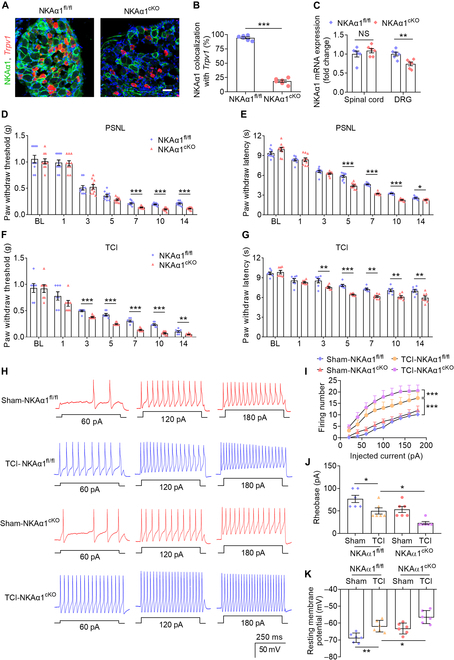
Conditional knockout of NKAα1 promotes pain sensation and DRG neuronal hyperexcitability in mice. (A and B) Representative images (A) and colocalization analyses (B) showing *Trpv1* (red) and NKAα1 (green) in DRG (*n* = 6). (C) qPCR analysis showing the mRNA expression of NKAα1 in the spinal cord and DRG of NKAα1^fl/fl^ and NKAα1^cKO^ mice (*n* = 6). (D to G) Behavior tests showing PSNL-induced (*n* = 10) (D and E) or TCI-induced (*n* = 7 or 8) (F and G) mechanical allodynia and thermal hyperalgesia in NKAα1^fl/fl^ and NKAα1^cKO^ mice. (H to K) Representative action potential traces (H) and group data showing firing number (I), rheobase (J), and resting membrane potential (K) in the DRG of sham-NKAα1^fl/fl^, TCI-NKAα1^fl/fl^, sham-NKAα1^cKO^, and TCI-NKAα1^cKO^ mice (*n* = 6 cells from 3 mice). The scale bars in (A) indicate 50 μm. Data are presented as mean ± SEM. Statistical analysis by unpaired Student *t* test in (B) and (C) or 2-way analysis of variance (ANOVA) with Bonferroni’s post hoc test in (D) to (G) and (I) or one-way ANOVA with Bonferroni’s post hoc test in (J) and (K). NS, not significant; BL, baseline. *P < 0.05; **P < 0.01; ***P < 0.001.

Subsequently, we explored the role of NKAα1 in nociceptive sensory neurons in modulating PSNL or TCI-evoked pain behaviors [34,35]. Genetic ablation of TRPV1-positive nociceptors causes a complete loss of thermal pain sensitivity and partially affects behavioral responses to mechanical noxious stimuli [36,37]. These findings underscore the critical contribution of TRPV1^+^ nociceptors to the transmission of nociceptive signals. Accordingly, TRPV1-cre and NKAα1^fl/fl^ mice were used to generate conditional NKAα1 knockout mice (NKAα1^cKO^) in which NKAα1 is specifically deleted in DRG nociceptors (Fig. 3A and B and Fig. S2A). Compared to that in NKAα1^fl/fl^ mice, the mRNA expression of NKAα1 was decreased in the DRG of NKAα1^cKO^ mice, but not in the spinal cord (Fig. 3C). Furthermore, compared to NKAα1^fl/fl^ mice, NKAα1^cKO^ mice developed more severe mechanical allodynia and heat hyperalgesia in response to PSNL or TCI (Fig. 3D to G). Compared with the PSNL model, NKAα1^cKO^ mice exhibited more rapid alterations in mechanical and thermal sensitivity after TCI surgery, suggesting that NKAα1 contributes more critically to the development of BCP than to the pathogenesis of neuropathic pain. Therefore, we paid more attention to the function of NKAα1 in TCI mice.

Most of the TRPV1-positive nociceptors are small-diameter neurons, with TRPV1 playing a critical role in modulating the hyperexcitability of small-diameter neurons [38–41]. To assess neuronal excitability, we performed patch-clamp recordings on small-diameter (<25-μm) nociceptive DRG neurons isolated from either TCI or sham-treated mice. Our data demonstrated that TCI treatment elevated the firing rate of nociceptors, with an even greater increase observed in TCI-NKAα1^cKO^ mice (Fig. 3H and I). Furthermore, compared to those of sham-NKAα1^fl/fl^ mice, both the rheobase and the absolute value of resting membrane potential were reduced in TCI-NKAα1^fl/fl^ mice and further decreased in TCI-NKAα1^cKO^ mice (Fig. 3J and K). These findings indicate that NKAα1 knockout in nociceptors promotes pain hypersensitivity by modulating neuronal activities in mice.

### NKAα1 interacts with P2X3R and regulates P2X3R-dependent Ca^2+^ influx and pain-like behavior

**Fig. 4.**
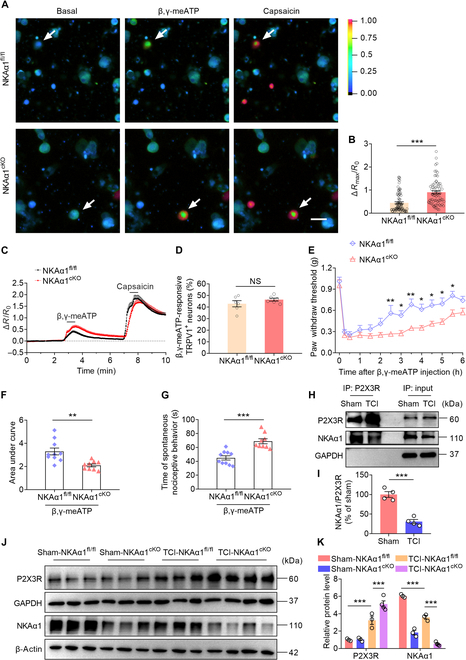
Impaired interaction between P2X3R and NKAα1 modulates P2X3R function in DRG neurons. (A to C) Representative images (A) and group data (B) showing Ca^2+^ responses and traces of time-dependent intracellular Ca^2+^ influx (C) induced by beta,gamma-methyleneadenosine 5′-triphosphate (β,γ-meATP) (100 μM) and capsaicin (1 μM) treatment. (D) Percentage of β,γ-meATP-responsive transient receptor potential vanilloid 1-positive (TRPV1^+^) neurons (defined as TRPV1^+^ neurons showing positive responses to capsaicin). Data represent 6 independent experiments per group, conducted on neurons derived from 2 independent cultures, with each experiment yielding 42 to 63 TRPV1^+^ responsive neurons. (E to G) Behavior tests showing the mechanical allodynia (E and F) and spontaneous pain-like behavior (G) after intraplantar injection of β,γ-meATP (10 nmol) in NKAα1^fl/fl^ and NKAα1^cKO^ mice (*n* = 9 or 10). (H and I) Co-immunoprecipitation analysis showing that the interaction between NKAα1 and P2X3R in DRG was reduced in TCI mice (*n* = 4). (J and K) Representative western blots and analyses of P2X3R and NKAα1 in the DRG of sham-NKAα1^fl/fl^, TCI-NKAα1^fl/fl^, sham-NKAα1^cKO^, and TCI-NKAα1^cKO^ mice (*n* = 3 or 4). The scale bars in (A) indicate 30 μm. Data are presented as mean ± SEM. Statistical analysis by unpaired Student *t* test in (B), (D), (F), (G), and (I) or 2-way ANOVA with Bonferroni’s post hoc test in (E) and (K). NS, not significant. *P < 0.05; **P < 0.01; ***P < 0.001.

We further investigate how NKAα1 knockout in TRPV1^+^ neurons promote the activities of DRG nociceptors and pain sensitivity of mice. Given the interaction between NKAα1 and P2X3R, we hypothesize that expression of NKAα1 in TRPV1^+^ neurons may modulate P2X3R function. To assess calcium mobilization, Ca^2+^ imaging was employed to examine beta, gamma-methyleneadenosine 5′-triphosphate (β,γ-meATP)-evoked responses in TRPV1^+^ neurons derived from both naive NKAα1^fl/fl^ and NKAα1^cKO^ mice. Consistent with our expectations, neurons lacking NKAα1 exhibited a markedly enhanced Ca^2+^ influx upon β,γ-meATP stimulation when compared to control NKAα1^fl/fl^ neurons (Fig. 4A to C). There is no difference in the TRPV1-specific agonist capsaicin-induced Ca^2+^ influx in DRG neurons from NKAα1^fl/fl^ and NKAα1^cKO^ mice (Fig. 4C). In addition, there was no difference in the proportion of TRPV1^+^ neurons responding to β,γ-meATP (Fig. 4D). These results suggest that NKAα1 knockout specifically enhances P2X3R-dependent Ca^2+^ influx. Furthermore, in both NKAα1^fl/fl^ and NKAα1^cKO^ mice, intraplantar injection of β,γ-meATP markedly induced mechanical allodynia, while the recovery of allodynia in NKAα1^cKO^ mice was much slower than that in NKAα1^fl/fl^ mice (Fig. 4E and F). Similarly, intraplantar injection of β,γ-meATP induced a pronounced spontaneous pain response 15 min postinjection, which was markedly enhanced in NKAα1^cKO^ mice (Fig. 4G), indicating enhanced P2X3R function in the DRG of these mice. Our results reveal that NKAα1 deficiency promotes P2X3R-dependent Ca^2+^ entry in TRPV1^+^ neurons, which contributes to alterations in pain-associated behaviors.

Similarly, we successfully constructed 3 plasmids, 1 for overexpressing NKAα1, 1 for overexpressing P2X3R, and 1 for knocking down NKAα1, and subsequently validated the transfection efficiency by analyzing both protein and mRNA expression levels (Fig. S2B to G). In addition, we observed Ca^2+^ imaging and found that knockdown of NKAα1 increased β,γ-meATP-induced calcium mobilization in HEK293T (human embryonic kidney 293T) cells overexpressing P2X3R, while overexpression of NKAα1 decreased calcium mobilization (Fig. S2H and I). We also used the P2X3R-selective antagonist A-317491 to pharmacologically block its function. Our results showed that inhibition of P2X3R abolished the effect of NKAα1 deficiency on pain sensitivity, while the analgesic effect of A-317491 persisted for over 6 h (Fig. S2J). These findings suggest that NKAα1 modulates P2X3R-dependent Ca^2+^ influx and that once P2X3R function is lost, NKAα1 no longer influences mechanical allodynia in mice.

To further investigate the reasons for the increased sensitivity of nociceptors in NKAα1^cKO^ mice after TCI treatment, a Co-IP experiment was utilized. It was found that the interaction between NKAα1 and P2X3R was reduced after TCI (Fig. 4H and I), suggesting an impaired modulation by NKAα1 on P2X3R function. In addition, compared to that in the sham-NKAα1^fl/fl^ group, the protein expression of P2X3R had no obvious change in the sham-NKAα1^cKO^ group. However, the expression of P2X3R was increased in the TCI-NKAα1^fl/fl^ group and further aggravated in the TCI-NKAα1^cKO^ group (Fig. 4J and K). These results indicate that NKAα1 knockout had no impact on P2X3R levels under sham conditions, yet it enhanced P2X3R expression following TCI treatment.

To further explain the reduced interaction between NKAα1 and P2X3R, along with the increased expression of P2X3R, we analyzed the expression of protein phosphatase 2A (PP2A), previously shown to enhance the membrane localization of NKAα1 by dephosphorylating serine residues on the NKA subunit [21,42,43]. The results showed that PSNL and TCI treatments markedly reduced the protein expression of PP2A (Fig. S3A to D), which partly explains the reduced interaction between NKAα1 with P2X3R. Previous research has demonstrated that within a BCP model, the increase in P2X3R levels in DRG is facilitated by the activation of the extracellular signal-regulated kinase 1/2 (ERK1/2) and Runt-related transcription factor 1 (Runx1) signaling cascade [9]. Considering that activated PP2A can dephosphorylate ERK1/2 [44], we hypothesized that the decreased PP2A expression could promote P2X3R expression by activating ERK1/2–Runx1 signaling (Fig. S3E). In line with earlier reports, our data revealed increased expression of phosphorylated ERK1/2 and Runx1 proteins in the TCI group relative to that in the sham group (Fig. S3F to I). The findings indicate that TCI treatment enhances P2X3R expression by activating ERK1/2–Runx1 signaling. PP2A is sensitive to Ca^2+^ signaling, and PP2A is phosphorylated and inactivated by calcium/calmodulin-dependent protein kinase II [45]. Thus, we speculate that Ca^2+^ influx was further elevated in the TCI-NKAα1^cKO^ group relative to that in the TCI-NKAα1^fl/fl^ group during the progression of TCI treatment, accompanied by activation of ERK1/2–Runx1 signaling. This could account for the enhanced elevation of P2X3R expression observed in TCI-NKAα1^cKO^ mice relative to that in TCI-NKAα1^fl/fl^ mice (Fig. 4J and K). These results suggest that NKAα1 directly affects the function of purinergic P2X receptors rather than their expression. In addition, we propose that NKAα1 dysfunction-induced intracellular Ca^2+^ influx activates ERK1/2–Runx1 signaling, thereby indirectly promoting P2X3R expression.

In summary, these findings suggest that TCI down-regulates the NKAα1 protein level and impairs the complex between NKAα1 and P2X3R in DRG, which facilitates P2X3R-mediated calcium mobilization and nociceptor excitability, and thereby induces pain-like behavior in TCI mice.

### Conditional knockout of NKAα1 exacerbates TCI-induced nerve injury and chemokine CCL5 release

**Fig. 5.**
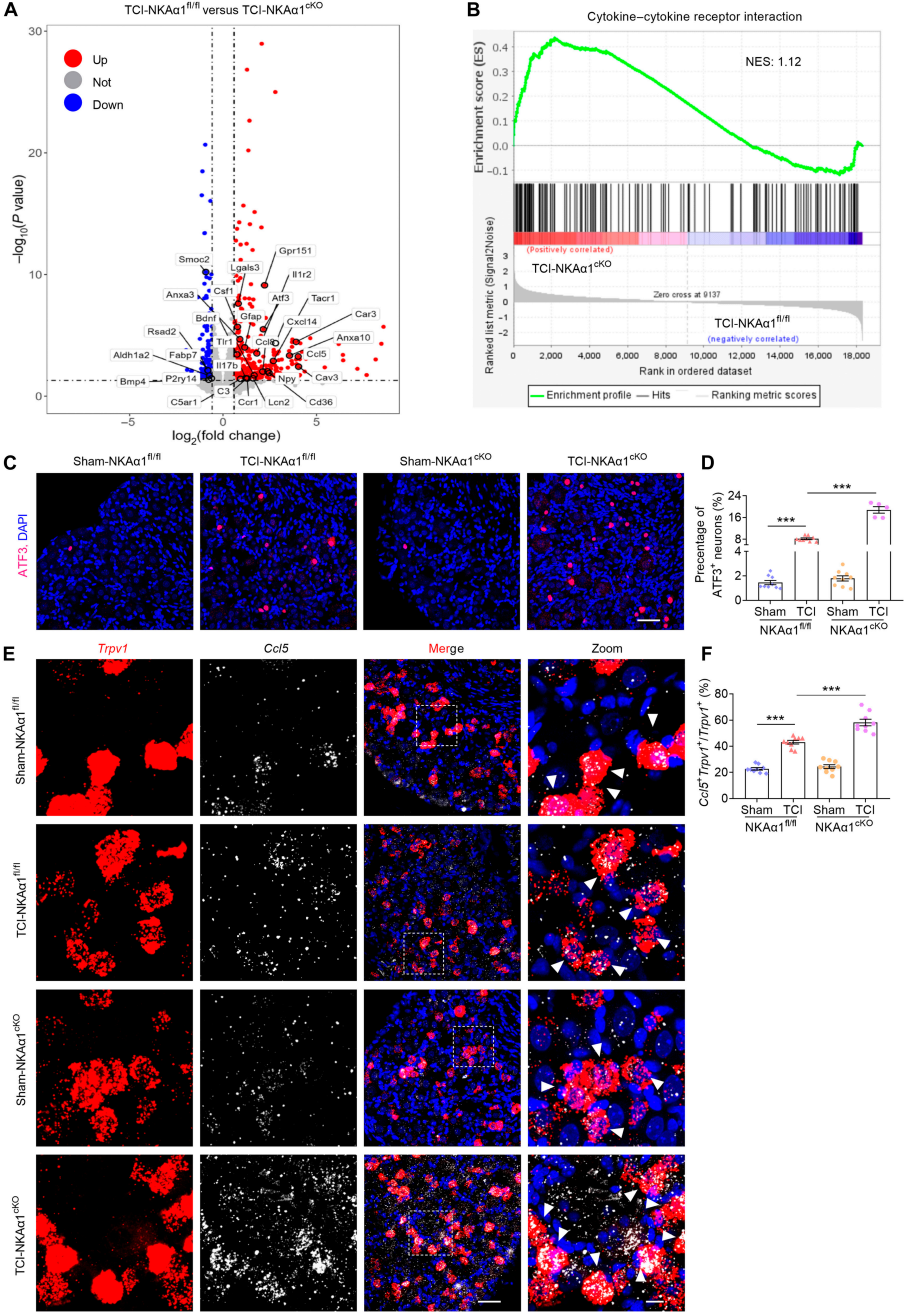
Conditional knockout of NKAα1 promotes TCI-induced DRG nerve injury and C-C motif chemokine ligand 5 (CCL5) release. (A) Volcano map of differentially expressed genes (DEGs) determined by whole-transcriptome RNA sequencing of DRG tissues from 3-month-old TCI-NKAα1^fl/fl^ and TCI-NKAα1^cKO^ mice. Normalized expression values (up, red; down, blue) were calculated for each DEG. (B) Gene set enrichment analyses (GSEAs) of upregulated genes in the DRG from TCI-NKAα1^fl/fl^ and TCI-NKAα1^cKO^ mice. (C and D) Representative images (C) and group data (D) showing the activating transcription factor 3 (ATF3; red) and 4′,6-diamidino-2-phenylindole (DAPI; blue) in DRG from sham-NKAα1^fl/fl^, TCI-NKAα1^fl/fl^, sham-NKAα1^cKO^, and TCI-NKAα1^cKO^ mice (*n* = 5 or 9). (E and F) Representative images (E) and group data (F) showing the colocalization of *Trpv1* (red) and *Ccl5* (white) in DRG from sham-NKAα1^fl/fl^, TCI-NKAα1^fl/fl^, sham-NKAα1^cKO^, and TCI-NKAα1^cKO^ mice (*n* = 9). The scale bars in (C) indicate 30 μm. The scale bars in (E) indicate 50 μm (left) and 10 μm (right). Data are presented as mean ± SEM. Statistical analysis by one-way ANOVA with Bonferroni’s post hoc test in (D) and (F). ***P < 0.001. NES, normalized enrichment score.

To further investigate the effect of NKAα1 knockout in TRPV1^+^ neurons on the TCI model, RNA sequencing (RNA-seq) was performed using acutely harvested DRG samples from TCI-NKAα1^fl/fl^ and TCI-NKAα1^cKO^ mice (Fig. S3J). Differential expression analysis identified 568 genes in TCI-NKAα1^cKO^ mice, of which 403 were upregulated and 165 were down-regulated (*P* < 0.05, fold change > 1.5) (Data S2). Notably, numerous genes associated with nociception and inflammation were markedly elevated, including chemokine-related genes (*Ccl5*, *Ccl8*, and *Cxcl14*), complement system (*C3* and *C5ar1*), and pain-relevant genes (*Lcn2*, *Lgals3*, and *Atf3*) (Fig. 5A and Fig. S3J). According to Kyoto Encyclopedia of Genes and Genomes pathway analysis, the upregulated gene set was markedly enriched in pathways related to cytokine–cytokine receptor interaction and calcium signaling pathway (Fig. S3K). Furthermore, gene set enrichment analyses (GSEAs) demonstrated a marked increase in cytokine–cytokine receptor interaction signatures in the DRG of TCI-NKAα1^cKO^ mice (Fig. 5B). Collectively, these results imply that NKAα1 knockout promotes activation of inflammatory responses and calcium signaling pathways in DRG.

Activating transcription factor 3 (ATF3), widely regarded as a marker of nerve cell injury [24,46], was utilized to assess the neuronal damage in DRG after NKAα1 knockout. Our findings demonstrated a marked elevation in the number of ATF3-positive (ATF3^+^) neurons following TCI treatment and it was further increased in TCI-NKAα1^cKO^ mice (Fig. 5C and D), which is consistent with RNA-seq analyses. These results suggest that NKAα1 knockout further promotes TCI-induced TRPV1^+^ neuronal damage. Evidence from spared nerve injury and PSNL models suggests that colony-stimulating factor 1 (CSF1) and galectin-3 (*Lgals3*), secreted by damaged sensory neurons, drive microglial activation in the spinal cord and promote neuropathic pain [47,48]. In addition, multiple studies have suggested that CCL5 mediates neuropathic pain in rodent animals [49–51]. We did observe a less than 2-fold increase in *Csf1* and *Lgals3* in TCI-NKAα1^cKO^ mice compared to that in TCI-NKAα1^fl/fl^ mice, but an approximately 16-fold increase in *Ccl5* (Fig. 5A). Therefore, we speculate that NKAα1 knockout aggravates DRG neuronal damage, which promotes CCL5 release and mediates pain hypersensitivity in mice.

We utilized in situ hybridization experiments to examine this assumption. Our findings demonstrate that *Ccl5* was distributed in TRPV1^+^ neurons after TCI and was further increased in TCI-NKAα1^cKO^ mice (Fig. 5E and F), which reconfirms that NKAα1 deficiency promotes CCL5 release. We next evaluated the effects of pharmacological activation of P2X3R and TRPV1 on CCL5 release in DRG neurons. Compared with that in the NKAα1^fl/fl^ group, β,γ-meATP-induced P2X3R activation markedly enhanced CCL5 release in the NKAα1^cKO^ group, whereas capsaicin-induced TRPV1 activation did not elicit a similar effect (Fig. S3L and M). These findings indicate that the enhanced CCL5 release in NKAα1^cKO^ mice is specifically linked to P2X3R activation rather than TRPV1 signaling.

**Fig. 6.**
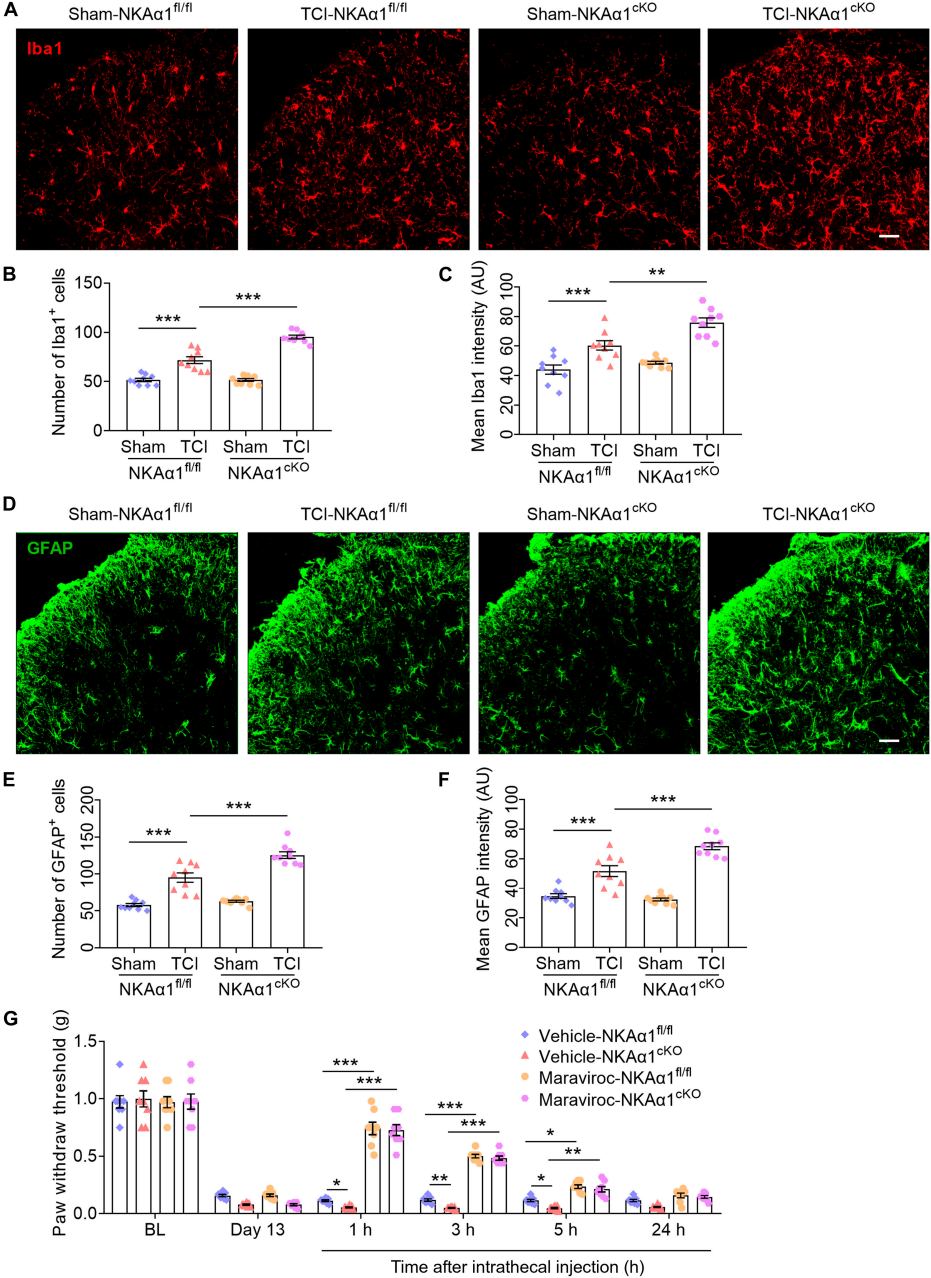
Conditional knockout of NKAα1 promotes TCI-induced spinal glial cell activation through the CCL5–C–C chemokine receptor type 5 (CCR5) axis. (A to C) Representative images (A) and group data showing the number of microglia (B) and mean intensity (C) of Iba1 (microglia marker, red) in DRG from sham-NKAα1^fl/fl^, TCI-NKAα1^fl/fl^, sham-NKAα1^cKO^, and TCI-NKAα1^cKO^ mice (*n* = 9). (D to F) Representative images (D) and group data showing the number of astrocytes (E) and mean intensity (F) of glial fibrillary acidic protein (GFAP; astrocyte marker, green) in DRG from sham-NKAα1^fl/fl^, TCI-NKAα1^fl/fl^, sham-NKAα1^cKO^, and TCI-NKAα1^cKO^ mice (*n* = 9). (G) von Frey test showing the time course of mechanical allodynia after single intrathecal injection of maraviroc (50 μg dissolved in saline) or vehicle in NKAα1^fl/fl^ and NKAα1^cKO^ mice after TCI treatment for 13 d (*n* = 8). The scale bars in (A) and (D) indicate 50 μm. Data are presented as mean ± SEM. Statistical analysis by one-way ANOVA with Bonferroni’s post hoc test in (B), (C), (E), and (F) or 2-way ANOVA with Bonferroni’s post hoc test in (G). *P < 0.05; **P < 0.01; ***P < 0.001.

The CCL5–C–C chemokine receptor type 5 (CCR5) axis has been shown to mediate a variety of pain hypersensitivity including BCP [49,52–54]. CCR5 was highly expressed in microglia, astrocytes, and monocytes [55,56]. We observed that the number and fluorescence intensity of microglia and astrocytes were increased in the spinal dorsal horn (SDH) after TCI and further increased in TCI-NKAα1^cKO^ mice (Fig. 6A to F). These results suggest that NKAα1 knockout promotes glial activation in the SDH. Maraviroc, a CCR5 blocker, relieved neuropathic pain in rodents when administered intrathecally [49,57]. A single intrathecal administration of maraviroc was found to markedly reduce TCI-induced mechanical allodynia, and the analgesic effect lasted for 5 h (Fig. 6G). These results suggest that inhibition of CCR5 improves pain perception. Together, NKAα1 knockout aggravates the release of CCL5 by TRPV1^+^ neurons, facilitating the activation of SDH microglia and astrocytes by binding on their CCR5, and aggravates pain hypersensitivity in mice.

### DR5-12D alleviates TCI-induced pain-like behavior by inhibiting DRG neuronal hyperexcitability

**Fig. 7.**
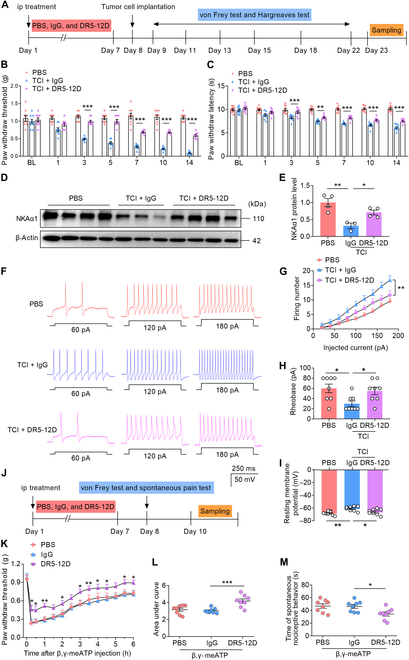
DR5-12D ameliorates TCI-induced pain-like behavior and DRG neuronal hyperexcitability. (A) Schematic of the experimental design. After 7 d of intraperitoneal (ip) injection of DR5-12D or IgG, mice were subjected to TCI, and then the von Frey test and Hargreaves test were performed. (B and C) Behavior tests showing TCI-induced mechanical allodynia (B) and thermal hyperalgesia (C) with or without receiving DR5-12D treatment (*n* = 9 or 10). (D and E) Representative western blots (D) and analyses (E) of NKAα1 in the DRG of PBS, TCI + IgG, and TCI + DR5-12D mice (*n* = 3 or 4). (F to I) Representative action potential traces (F) and group data showing firing number (G), rheobase (H), and resting membrane potential (I) in the DRG of PBS, TCI + IgG, and TCI + DR5-12D mice (*n* = 8 cells from 4 mice). (J) Schematic of the experimental design. After 7 d of intraperitoneal injection of DR5-12D or IgG, mice were subjected to the von Frey test and spontaneous pain test. (K to M) Behavior tests showing the mechanical allodynia (K and L) and spontaneous pain-like behavior (M) after intraplantar injection of β,γ-meATP (10 nmol) in PBS, IgG, and DR5-12D mice (*n* = 8). Data are presented as mean ± SEM. Statistical analysis by one-way ANOVA with Bonferroni’s post hoc test in (E), (H), (I), (L), and (M) or 2-way ANOVA with Bonferroni’s post hoc test in (B), (C), (G), and (K). *P < 0.05; **P < 0.01; ***P < 0.001.

A monoclonal antibody, DR5-12D, was previously generated in our laboratory to target the DR region of NKA, thereby enhancing the stability of the NKA protein [14,21,58]. To evaluate the safety of NKAα1 antibody therapy, wild-type mice were intraperitoneally injected with phosphate-buffered saline (PBS), control immunoglobulin G (IgG), or DR5-12D for 7 consecutive days, and cardiac and renal functions were assessed by electrocardiography and serum electrolyte analysis. The results showed that DR5-12D treatment did not induce marked alterations in either electrocardiographic parameters or serum electrolyte levels (Fig. S4A to H). Based on these findings, DR5-12D was subsequently applied in the BCP model, where mice received a 7-d pretreatment regimen prior to TCI induction, followed by behavioral assessment of mechanical and thermal nociception starting on day 9 (Fig. 7A). Compared with TCI mice treated with IgG, those receiving DR5-12D treatment exhibited marked relief from mechanical allodynia and thermal hyperalgesia starting on day 3 post-TCI, with the analgesic effects persisting until day 14 (Fig. 7B and C). These findings indicate that administration of DR5-12D effectively attenuates TCI-induced pain-like behavior. Western blots also showed that DR5-12D treatment substantially increased the protein level of NKAα1 (Fig. 7D and E). Moreover, DR5-12D treatment markedly attenuated TCI-induced hyperexcitability in DRG nociceptors, indicated by fewer action potentials, increased rheobase, and hyperpolarized resting membrane potential relative to those in the TCI-IgG group (Fig. 7F to I). Intraplantar injection of β,γ-meATP substantially induced mechanical allodynia in all groups, but the allodynia threshold was markedly higher in DR5-12D mice than in PBS or IgG mice (Fig. 7J to L). Similarly, administration of β,γ-meATP into the paw elicited marked spontaneous pain-related behaviors, although this response was attenuated in DR5-12D mice (Fig. 7M). These results indicate that DR5-12D alleviates TCI-induced pain-like behavior, potentially through maintaining the stability of NKAα1 protein and suppressing the hyperexcitability of nociceptive sensory neurons.

## Discussion

**Fig. 8.**
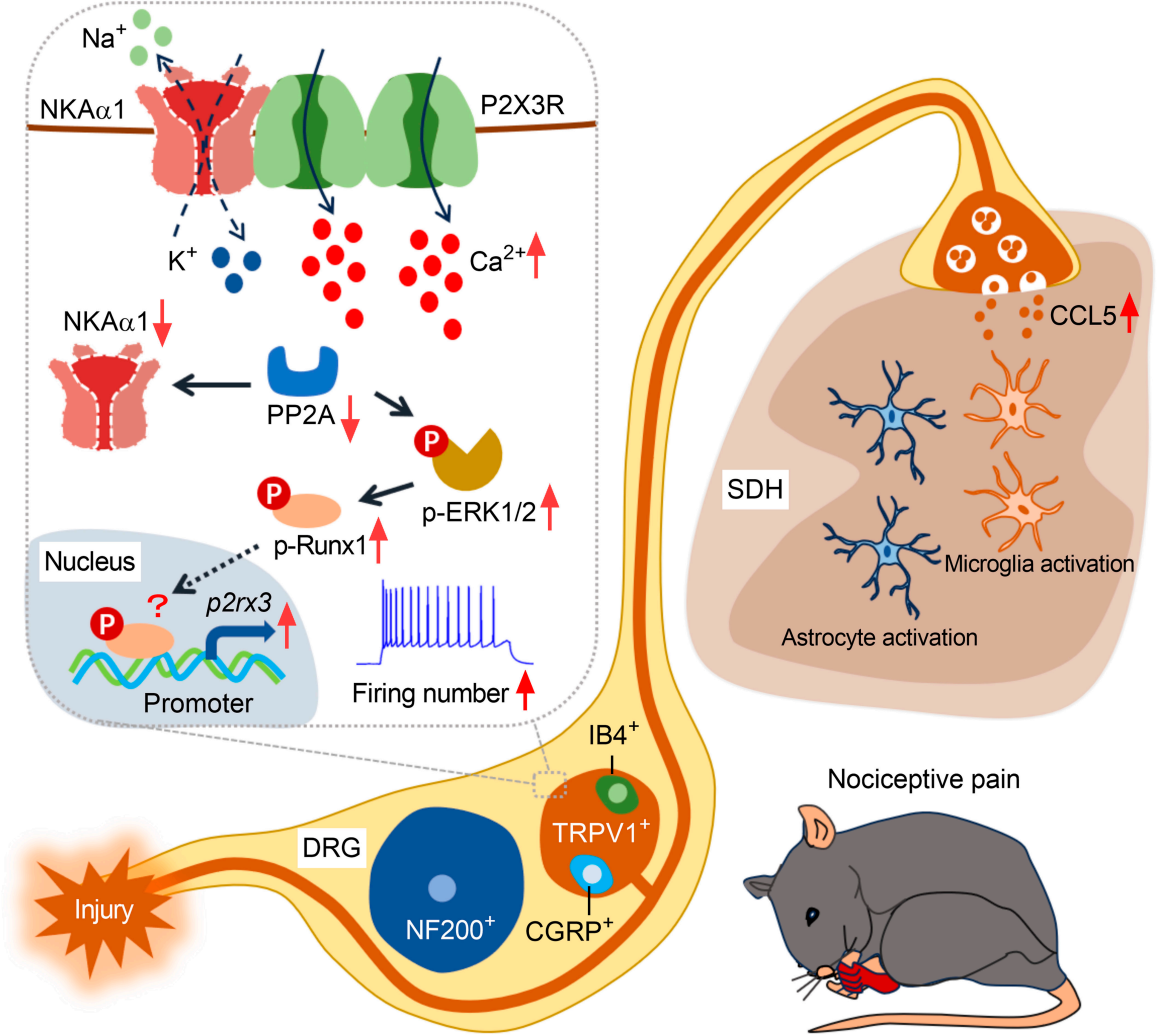
Schematic diagram illustrating the proposed mechanism in the TCI model: injury-induced activation of P2X3R channels leads to Ca^2+^ influx, which down-regulates protein phosphatase 2A (PP2A) expression and diminishes membrane localization of NKAα1. The decreased membrane NKAα1 disrupts its interaction with P2X3R, further promoting Ca^2+^ influx and additional suppression of PP2A. Concurrently, reduced PP2A expression activates the extracellular signal-regulated kinase 1/2 (ERK1/2)–Runt-related transcription factor 1 (Runx1) signaling pathway, upregulating P2X3R expression. Together, these changes enhance the excitability of neurons and neuronal injury, which in turn promotes CCL5 release and glial cell activation, ultimately inducing bone cancer pain. SDH, spinal dorsal horn.

NKA functions as an ATP-dependent ion transporter that preserves intracellular ionic balance and contributes critically to cellular physiological activities [14,59,60]. This study aimed to elucidate the specific mechanisms governing Ca^2+^ homeostasis in DRG neurons that underlie TCI-induced pain-like behavior. We found that NKAα1 modulates P2X3R-dependent Ca^2+^ influx, thereby regulating nociceptive pain-like behavior in mice. To our knowledge, this work provides the first evidence revealing the role of NKAα1 in P2X3R-dependent Ca^2+^ influx and the excitability of nociceptors. We observed that DR5-12D antibody demonstrated marked efficacy in alleviating BCP (Fig. 8). As a result, NKAα1 is proposed as a key modulator of P2X3R-dependent Ca^2+^ homeostasis and a promising intervention point for alleviating BCP.

Activated by ATP, purinergic P2X receptors act as nonselective cation channels, mediating Na^+^ and Ca^2+^ influx alongside K^+^ efflux and display particularly high permeability to Ca^2+^ [61]. Emerging studies underscore the pivotal involvement of purinergic P2X receptors in cancer pain mechanisms [23], especially P2X3R, P2X4R, and P2X7R [62]. Furthermore, P2X4R and P2X7R are mostly expressed in immune cells, while P2X3R is mostly expressed in neurons [23,63], which are consistent with our results. Furthermore, growing evidence supports the idea that the Ca^2+^ signaling pathway participates in BCP [64,65]. However, the primary function of NKA is to exchange intracellular Na^+^ with extracellular K^+^ [66]. Therefore, we propose that NKA can also indirectly modulate Ca^2+^ transport. In the present study, we did observe an increased Ca^2+^ response to P2X3R in the DRG neurons of NKAα1^cKO^ mice. Previous research on oligodendrocytes has provided a possible explanation for this phenomenon, with NKA blockade resulting in elevated intracellular Na^+^ levels, which may cause a shift of the Na^+^–Ca^2+^ exchanger (NCX) reversal potential toward more negative values, thereby potentially triggering NCX-mediated Ca^2+^ influx [67]. Thus, it is possible that NKA dysfunction mediates Ca^2+^ influx through NCX and promotes TCI-induced pain-like behavior.

Previous research has demonstrated that ASIC3 forms a protein complex with P2X3R and promotes inflammatory pain [25]. Similarly, GPR151 couples with P2X3R, thereby enhancing their contribution to neuropathic pain signaling [24]. Unlike previous studies, our current findings suggest that the interaction between NKAα1 and P2X3R inhibits P2X3R-dependent Ca^2+^ influx and pain hypersensitivity in mice. Moreover, conditional knockout of NKAα1 in TRPV1^+^ neurons further promoted neuronal hyperexcitability and pain hypersensitivity in mice. In turn, DR5-12D stabilized the expression of NKAα1, markedly inhibited TRPV1^+^ neuronal hyperexcitability, and ameliorated pain hypersensitivity in mice. Similarly, our previous study showed that stabilizing the NKAα1–P2X7R complex in microglia inhibited P2X7R-mediated K^+^ efflux, thereby ameliorating microglial activation and alleviating anxiety-like behavior in mice [21]. These results suggest that NKAα1 may modulate the different ion transport function of the purinergic P2X receptors to some extent.

Sensory neurons are able to produce cytokines and chemokines in response to nerve injury [68], such as CCL2, CSF1, galectin-3, and interleukin-6 [47,48,69,70], and facilitate glial activation within the spinal cord. Evidence from this study suggests that injured TRPV1^+^ neurons released CCL5 and promoted the activation of microglia and astrocytes in the SDH, which was exacerbated by NKAα1 knockout. Studies have shown that activation of the CCL5–CCR5 axis mediates paclitaxel-induced peripheral neuropathic pain or herpes-related neuralgia, while blockade of CCR5 has an evident analgesic effect [49,57]. Similarly, our results also showed that blockade of CCR5 markedly improved TCI-induced pain-like behavior. A transcription factor rapidly upregulated following injury, ATF3 plays a crucial role after injury and is essential for neuronal functional recovery [71]. In addition, ATF3 activation has been shown to induce chemokine secretion [72]. Previous studies have indicated that activation or upregulation of ATF3 is closely linked to epigenetic changes, particularly enhanced histone acetylation such as increased H3K27ac enrichment at the ATF3 promoter, which facilitates its transcriptional activation [73]. Notably, TCI has been shown to contribute to the progression of BCP by elevating H3K27ac levels at the GPR160 promoter region [74]. In addition, ATF3 has been identified as a transcriptional regulator of CCL5 [75]. In this study, ATF3 expression was markedly increased in the TCI-NKAα1^cKO^ group compared to that in the TCI-NKAα1^fl/fl^ group. This elevation might result from TCI-induced alterations in histone acetylation at the ATF3 locus, promoting ATF3 expression, which subsequently upregulates CCL5 and contributes to the development of BCP.

Previous research has indicated that the peripheral mechanisms underlying BCP involve a complex interplay of inflammatory and neuropathic components, including the activation of nociceptors by soluble factors released from cancer cells, tumor-associated immune cells, and osteoclasts [76,77]. Building on this, we hypothesize that NKAα1 expressed in TRPV1^+^ neurons may contribute not only to neuropathic pain resulting from nerve injury but also to additional pathological processes involved in BCP in the TCI mouse model. Consistent with this hypothesis, our results demonstrated that the influence of NKAα1 on BCP-related behaviors was more pronounced than its effect on neuropathic pain. Nevertheless, further investigations are warranted to elucidate the underlying mechanisms responsible for the observed differences between these 2 models.

Although numerous studies have demonstrated that targeting P2X3R is an effective strategy for treating pain and several P2X3R antagonists, such as AF219, AF353, and A-317491, have undergone clinical testing, substantial progress in clinical applications has yet to be achieved [78]. In contrast, P2X3R antagonists can be clinically used to treat chronic obstructive pulmonary disease, asthma, and chronic cough [79–81]. This difference may be attributed to the distinct roles that P2X3R plays in 2 types of diseases. Pain responses are believed to be linked to P2X3R-dependent calcium fluctuations [78], whereas cough is associated with P2X3R-mediated inflammatory signaling [79]. Intracellular Ca^2+^ influx is influenced by multiple factors, which may result in limitations in the clinical application of targeting P2X3R for pain treatment. By using DR5-12D to stabilize the membrane expression of NKA, we observed that it exerts a marked analgesic effect. Therefore, targeting and inhibiting P2X3R-dependent Ca^2+^ influx at their source can serve as an effective therapeutic strategy to block pain sensation.

Our previous study demonstrated that NKAα1 exhibits anti-inflammatory effects in the brain [21]; similarly, in this study, we found that NKAα1 can counteract inflammation-induced BCP, indicating that NKAα1 could be considered a critical regulator in the neuroimmune system. This study is consistent with our previous findings that NKAα1 functions not merely as an ion pump but also as a pivotal cytoprotective protein [14,58]. In our previous and current studies, we observed reduced NKAα1 expression in mice with chronic stress-induced anxiety, in the BCP model, and in high-fat-diet-induced obesity [14,21,82]. These results suggest that diminished NKAα1 levels are a key driver of these disease phenotypes.

## Materials and Methods

### Study design

The purpose of this study was to investigate the involvement of NKAα1 in primary sensory neurons throughout the progression of BCP. We first characterized the distribution of NKAα1 in DRG and assessed whether its deletion modulates neuronal hyperexcitability and BCP-related behaviors. We then explored the underlying mechanism and demonstrated that NKAα1 colocalizes with P2X3R in DRG neurons and that loss of NKAα1 enhances P2X3R function and P2X3R-mediated nociceptive responses. Finally, we evaluated the translational potential of these findings by examining the analgesic effect of DR5-12D antibody, which stabilizes NKAα1 expression.

### Ethics statement

All animal experiments received approval from the Institutional Animal Care and Use Committee at Southern University of Science and Technology (SUSTech-JY202012002) and were performed following established ethical standards for animal welfare.

### Animals

C57BL/6J mice (6 weeks old) were purchased from GemPharmatech. BALB/cJ and TRPV1-Cre mice (6 to 8 weeks of age) were purchased from The Jackson Laboratory, while NKAα1^fl/fl^ mice were custom-generated and confirmed by Cyagen Biosciences. To establish the conditional knockout (NKAα1^cKO^) model, TRPV1-Cre mice were bred with NKAα1^fl/fl^ mice. Unless stated otherwise, experiments were performed using male mice, which were randomly allocated to different groups. All mice were maintained in a specific-pathogen-free environment and kept on a 12-h light/dark schedule (lights on at 0700), at a stable ambient temperature of 23 to 25 °C, with unrestricted access to standard chow and water.

### Animal drug administration

Maraviroc (MCE, HY-13004) and A-317491 (MCE, HY-15568) were dissolved in saline and administered to mice via a single intrathecal injection, followed by subsequent behavioral testing.

### Cell culture

HEK293T cells (Horizon Discovery, HCL4517), verified by the supplier, were grown in Dulbecco’s modified Eagle medium (Dakewe, 6016121) supplemented with 10% heat-inactivated fetal bovine serum (NEST Biotechnology, 209111) and 1% penicillin–streptomycin (NEST Biotechnology, 211092). Cells were maintained at 37 °C in a 5% CO_2_-humidified atmosphere.

### Experimental model of tumor-induced bone pain

To establish a model of BCP, we introduced LLC1 tumor cells into the tibial cavity following a previously described procedure [83] with slight modifications. Prior to implantation, LLC1 cells were enzymatically dissociated using 0.25% trypsin, followed by resuspension in PBS (Procell, PB180327) with a target concentration of 1 × 10^8^ cells/ml. Following isoflurane anesthesia, a short incision (approximately 0.5 to 1.0 cm) was made beside the knee to uncover the patellar ligament. A 25-gauge microsyringe (Hamilton, USA) containing 2 μl of the LLC1 cell suspension was then inserted through the intercondylar notch, reaching the tibial marrow cavity. The suspension was slowly injected over a 2-min period to ensure adequate dispersion. In the control group, the same volume of PBS was administered using an identical protocol. Following the injection, the incision was closed with silk sutures and the area disinfected with sterile gauze.

### Neuropathic pain model

To establish peripheral neuropathic pain in mice, we employed a modified PSNL model, based on a previously established method [84] and further optimized in our laboratory. Mice were deeply anesthetized with isoflurane mixed with oxygen, followed by a small incision to reveal the left sciatic nerve. The dorsal half of the nerve was gently ligated using a 4–0 silk thread. After ligation, the muscle and skin were closed with sutures, and an antibiotic ointment was applied to the incision area.

### von Frey test

Building upon earlier studies, we refined the von Frey test protocol to improve its applicability [85]. Mice were acclimated for 30 min in separate chambers situated on an elevated metal mesh platform with controlled temperature and humidity. Mechanical sensitivity was evaluated using von Frey filaments (Stoelting, USA) exerting logarithmically increasing forces between 0.02 and 2.56 g, applied perpendicular to the plantar surface of the hind paw. Each filament was pressed for up to 2 s, and a swift paw withdrawal or licking was considered a positive reaction. The paw withdrawal threshold was determined through the up-down method to quantify mechanical allodynia. A minimum interval of 5 min was maintained between consecutive stimuli to avoid sensitization. Following 14 d of behavioral testing, DRG tissues were collected for subsequent analyses.

### Hargreaves test

Thermal pain sensitivity was measured using the Hargreaves apparatus. Individual mice were placed in clear Plexiglas chambers on a glass plate kept at 30 °C. An adjustable infrared heat source was directed onto the plantar surface of each hind paw. Each paw underwent 3 trials with a minimum interval of 5 min between tests. To avoid tissue injury, a maximum exposure time of 20 s was enforced. Fourteen days after thermal testing, DRG tissues were collected for further analysis.

### Spontaneous pain test

For evaluation of spontaneous pain behavior, mice were individually housed in clear Plexiglas enclosures set on a raised metal mesh platform for 10 min. Behavioral activity was continuously recorded using a video camera. Prior to testing, animals were acclimated to the environment for 30 min. The final 10 min of the recordings was subsequently analyzed offline by an experimenter blinded to the treatment groups. The cumulative time spent licking, lifting, shaking, or guarding the affected hind paw was quantified as an indicator of spontaneous pain [86].

### Plasmid construction and transient transfection

Three different plasmids were constructed, namely, pCDH-CMV-*ATP1a1*-3×HA-EF1a-CopGFP-T2A-PuroR-WPRE, pcDNA3-CMV-mRuby3-*P2rx3*-WPRE, and pLOK.1-U6-sh*Atp1a1*-hPGK-PuroR-WPRE. They were used for overexpression of *ATP1a1* and *P2rx3* as well as knockout of *Atp1a1*, respectively. HEK293T cells were plated into 6-well plates and transfected according to the Lipofectamine 3000 (Invitrogen, L3000015) protocol provided by the manufacturer. For single-gene expression, cells underwent transfection with 2 μg of either *P2rx3* or *ATP1a1* plasmid. For co-expression studies, 1 μg of each plasmid (*P2rx3* and *ATP1a1*) was co-transfected. In a separate group, cells received 1 μg of sh*ATP1a1* plasmid to achieve knockdown of *ATP1a1*. Cells were allowed to incubate for 48 h post-transfection prior to proceed with subsequent experiments.

### Primary culture of DRG neurons

Lumbar DRGs (L4 to L6) were harvested from young mice and promptly immersed in PBS. The tissues underwent enzymatic digestion at 37 °C for 60 min using a solution containing collagenase type IV and dispase II (Solarbio; C8160 or D6431). After digestion, cells were suspended in a neurobasal medium supplemented with 10% fetal bovine serum, 1% penicillin–streptomycin, and 2% B27. The isolated DRG neurons were then seeded onto glass coverslips coated with 1 mg/ml poly-d-lysine (Solarbio, D6790) for further culture and experimental procedures.

### Electrophysiological recordings of DRG neurons

Primary DRG neurons cultured on glass coverslips were placed in a recording chamber mounted on the fixed stage of an upright microscope (BX51WIF, Olympus, Japan). The chamber was continuously superfused with an external solution containing (mM) 140 NaCl, 5 KCl, 1 MgCl_2_, 2 CaCl_2_, 10 HEPES, and 10 d-glucose, adjusted to pH 7.4 with NaOH and osmolarity of 300 to 310 mOsm. Patch pipettes (3 to 6 MΩ) were filled with an internal solution consisting of (mM) 135 K-gluconate, 5 NaCl, 2 MgCl_2_, 10 HEPES, 0.5 EGTA, 2 MgATP, and 0.4 Na_2_GTP. Neurons were visualized using a ×40 water-immersion objective, and whole-cell patch-clamp recordings were conducted with a MultiClamp 700B amplifier (Molecular Devices, USA). To assess intrinsic excitability, stepwise depolarizing current pulses ranging from 0 to 180 pA were applied for 600 ms. Action potentials were elicited during current-clamp mode by injecting incremental currents, whereas resting membrane potential was recorded in the absence of current injection.

### Calcium imaging

Intracellular Ca^2+^ levels were monitored in cultured DRG neurons and HEK293T cells. To identify specific neuronal subtypes, β,γ-meATP (MCE, HY-134440A) was applied to mark P2X3R^+^ cells, and capsaicin (TargetMol, USA, T1062) for TRPV1^+^ cells, and 50 mM KCl served as a positive control. DRG neurons were loaded with 5 μM fura-2/AM (YEASEN, 40702ES72) in Ringer’s solution (130 mM NaCl, 2.5 mM CaCl_2_, 3 mM KCl, 0.6 mM MgCl_2_, 10 mM HEPES, and 10 mM glucose; pH 7.4) and incubated at 37 °C for 30 min. Fluorescence excitation wavelengths of 340 and 380 nm were alternated using a Lambda DG-5 rapid wavelength switcher (Sutter Instrument, USA). Images were acquired every 2 s and analyzed with the MetaFluor software (Molecular Devices). The intracellular calcium concentration ([Ca^2+^]_i_) was then determined through the computation of the emission intensity ratio between these 2 excitation wavelengths. Data are expressed as Δ*R*/*R*_0_, where Δ*R* = *R*_t_ − *R*_0_; cells with Δ*R*/*R*_0_ > 0.05 were classified as responsive.

### Co-IP performed in vivo

DRG tissues were homogenized in NP-40 lysis buffer (Absin, abs9160) supplemented with protease inhibitors and centrifuged at 12,000 × g for 10 min to clear debris. The resulting supernatant was collected, with 5% of the total lysate reserved as an input control and mixed with an equal volume of 2× Laemmli buffer for denaturation. For each Co-IP assay, 1,000 μg of total protein was incubated with either 2 μg of rabbit IgG (ABclonal, AC005) as a negative control or 2 μg of anti-P2X3R antibody (Abcam, ab300493). The reaction mixtures were incubated overnight at 4 °C with gentle rotation to promote antigen–antibody complex formation. Afterward, 50 μl of pre-washed Protein A/G magnetic beads (Absin, abs9649) were suspended in Co-IP buffer and added to the samples, followed by an additional 4 h of gentle rotation at 4 °C to capture immune complexes. Beads were then collected on a magnetic rack, washed 3 times with lysis buffer to remove nonspecific proteins, and boiled in 2× Laemmli buffer at 95 °C for 5 min to release bound proteins. The eluates were analyzed by sodium dodecyl sulfate–polyacrylamide gel electrophoresis (SDS-PAGE) and western blotting to detect the immunoprecipitated targets.

### Western blotting

DRG tissues were homogenized on ice for 5 min using a no-touch ultrasonic homogenizer (SCIENTZ08 IIIC, SCIENTZ, China) in radioimmunoprecipitation assay (RIPA) buffer containing a cocktail of protease inhibitors (Abbkine, BMP1001). The homogenates were centrifuged at 12,000 × g for 10 min at 4 °C, and the resulting supernatant was collected. For HEK293T cells, lysis was performed on ice for 30 min using RIPA buffer (Absin, abs9229), followed by centrifugation under identical conditions. Protein levels in all samples were determined with a bicinchoninic acid protein assay kit (Solarbio, PC0020). Equal amounts of protein (30 μg per lane) were separated by SDS-PAGE and transferred onto polyvinylidene fluoride membranes (0.45 μm, Millipore, IPVH00010). The membranes were first blocked at room temperature for 1 h in Tris-buffered saline with 0.1% Tween-20 containing 5% nonfat milk and then incubated overnight at 4 °C with the following primary antibodies: NKAα1 (1:1,000, Invitrogen, MA3-928), P2X3R (1:1,000, Abcam, ab300493), phosphorylated ERK1/2 (1:1,000, CST, #9102), phosphorylated Runx1 (1:200, Santa Cruz, sc-293146), PP2Ac (1:1,000, ABclonal, A6702), β-actin (1:5,000, Proteintech, 20536-1-AP), and glyceraldehyde-3-phosphate dehydrogenase (GAPDH; 1:5,000, New Cell & Molecular Biotech, AB2100). After washing, the membranes were incubated for 90 min at room temperature with horseradish peroxidase-labeled secondary antibodies (1:5,000, CST, #7074 or #7076). Protein signals were detected using chemiluminescence with Sparkjade ECL Plus (Shandong Sparkjade Biotechnology Co., Ltd., ED0016-C) and quantified using the ImageJ software.

### Immunohistochemistry

Before tissue collection, mice were anesthetized via intraperitoneal administration of sodium pentobarbital (40 mg/kg) and subsequently perfused transcardially with 0.9% saline, followed by 4% paraformaldehyde. The L4 to L6 DRG and corresponding spinal cord segments were harvested, postfixed in 4% paraformaldehyde at 4 °C for 24 h, and subsequently immersed in 30% sucrose at 4 °C for cryoprotection. Tissue samples were frozen and coronally sectioned at 15 μm for DRG and 30 μm for spinal cord using a Leica CM1900 cryostat. Sections were blocked for 2 h at room temperature in PBS supplemented with 5% donkey serum and 0.3% Triton X-100 and then incubated overnight at 4 °C with primary antibodies against P2X3R (1:100, Abcam ab300493), NKAα1 (1:200, Invitrogen MA3-928; 1:100, Proteintech 14418-1-AP), CGRP (1:300, Abcam ab47027), NF200 (1:200, Sigma N0142), TH (1:200, Abcam ab6211), ATF3 (1:100, Sigma HPA001562), Iba1 (1:800, Wako 019-19741), GFAP (1:500, Invitrogen MA5-12023), and TRPV1 (1:100, Sigma SAB5700857). After 3 washes with PBS containing 0.05% Tween-20, sections were incubated overnight at 4 °C with Alexa Fluor 488- or 594-conjugated donkey secondary antibodies (1:1,000, Invitrogen) or with IB4 lectin (1:100, Sigma L2895), depending on the primary marker. Following another 3 washes, nuclei were counterstained with 4′,6-diamidino-2-phenylindole (Coolaber, SL7101) for 10 min at room temperature. Fluorescence images were acquired on a Nikon A1R+Symp64 confocal microscope and analyzed with Fiji (ImageJ).

### In situ hybridization

DRG sections were subjected to single-molecule fluorescence in situ hybridization utilizing RNAscope Multiplex Fluorescent Reagent Kit (ACD, 323100). To improve probe accessibility, the tissues underwent sequential pretreatment with hydrogen peroxide, target retrieval buffer, and protease III, following the protocol provided by the manufacturer. Subsequently, the tissues were hybridized with specific probes targeting mouse *Atp1a1*, *Ccl5*, and *Trpv1* mRNAs. Signal amplification was achieved through sequential application of amplification reagents, and signal amplification and detection were achieved through horseradish peroxidase-linked probes combined with TSA Plus fluorophores—fluorescein isothiocyanate, Cy3, and Cy5—enabling multiplexed fluorescent visualization. Following hybridization, immunofluorescence staining was performed on tissue sections by incubating them with primary antibodies following standard procedures. To preserve fluorescent signals, all procedures were performed in the dark.

### DR5-12D monoclonal antibody production and preparation

As previously described [21], the DR5-12D monoclonal antibody was obtained from ascitic fluid collected from female BALB/cJ mice (6 to 8 weeks of age). For in vivo treatment, C57BL/6J mice received daily intraperitoneal injections of either the DR5-12D antibody or control IgG at a dose of 5 mg/kg for 7 d in succession.

### Quantitative real-time polymerase chain reaction

Total RNA from tissues and cells was isolated using TRIzol reagent (Invitrogen, 15596018CN) according to the manufacturer’s protocol. Complementary DNA was synthesized with ABScript Neo RT Master Mix for quantitative polymerase chain reaction (qPCR) with gDNA Remover (ABclonal, RK20433). qPCR was carried out using 2X Universal SYBR Green Fast qPCR Mix (ABclonal, RK21203), with GAPDH as the housekeeping gene for normalization. The relative transcript levels were determined by the 2^−ΔΔCT^ method. Primer sequences (Sangon Biotech, China) are provided in Table S1.

### RNA sequencing

Building on a previously reported method with minor adjustments [21,87], mice were anesthetized with sodium pentobarbital (40 mg/kg) and perfused transcardially with ice-cold saline. DRG tissues were then isolated in Hank’s balanced salt solution, minced into small pieces, and immediately subjected to RNA extraction using TRIzol reagent. RNA integrity was evaluated on Agilent Bioanalyzer 2100 [88] with RNA Nano 6000 Assay Kit (Agilent, 5067-1511). Libraries were prepared and sequenced on an Illumina NovaSeq platform to generate 150-bp paired-end reads. Adapter trimming was carried out with Cutadapt, and reads were mapped to the *Mus musculus* GRCm38 reference genome using HISAT2. Differential gene expression was assessed with DESeq2, applying an adjusted *P* value threshold of < 0.05. Enrichment analysis for Gene Ontology categories was performed with the clusterProfiler R package, and GSEA was executed using the GSEA software (v2.0.14).

### Reanalysis of scRNA-seq datasets

To investigate the cellular distribution of NKA subunits within DRG, we reanalyzed publicly available scRNA-seq datasets of the DRG [30]. To refine cell type classification and infer developmental trajectories, reference annotations and criteria from 2 previously published studies were incorporated into our analysis [89,90].

### Electrocardiographic recording and serum electrolyte analysis

Electrocardiographic signals were recorded in mice anesthetized with sodium pentobarbital (40 mg/kg) using the BL-420N physiological data acquisition system (Taimeng, China). Subcutaneous needle electrodes were positioned in the right forelimb (white), right hind limb (black), and left hind limb (red) and connected to channel 1; traces were acquired for ~3 min. For electrolyte analysis, blood was collected into heparinized tubes and centrifuged at 3,000 rpm for 15 min at 4 °C within 30 min of collection, and serum electrolytes were measured immediately on a Chemray 800 automated analyzer (Shenzhen Rayto, China).

### CCL5 enzyme-linked immunosorbent assay

DRG tissues were isolated from young mice for primary neuronal culture. Cultured DRG neurons were stimulated with β,γ-meATP or capsaicin for 90 min. Following treatment, culture supernatants were collected and subjected to enzyme-linked immunosorbent assay to measure CCL5 levels (Sigma, RAB0077) according to the manufacturer’s instructions.

### Mass spectrometric analysis via liquid chromatography tandem technique (LC–MS/MS)

Lyophilized peptide samples were dissolved in double-distilled water supplemented with 0.1% formic acid prior to analysis. A 2-μl portion of each sample was loaded onto a nanoViper C18 trap column (Acclaim PepMap 100, Thermo Fisher) for initial concentration and desalting, during which 20 μl of solvent A (0.1% formic acid in water) was applied. Peptides were then separated on an EASY-nLC 1200 system (Thermo Fisher, USA) using an analytical C18 column (Acclaim PepMap RSLC, Thermo Fisher) with linear gradient elution over 120 min, increasing solvent B (80% acetonitrile with 0.1% formic acid) from 5% to 38%. Mass spectrometric detection was performed on a Q Exactive Orbitrap instrument (Thermo Fisher, USA) equipped with a Nano Flex ion source. The spray voltage was set to 1.9 kV, and the interface heater temperature was maintained at 275 °C. Full MS spectra were acquired across an *m*/*z* range of 350 to 2,000 at a resolution of 70,000, using an automatic gain control target of 3 × 10^6^ and a maximum injection time of 100 ms. For MS/MS analysis, precursor ions with charge states of 2 to 5 were fragmented via higher-energy collisional dissociation at a normalized collision energy of 28. The resulting MS2 spectra were recorded in rapid scan mode within the ion trap, with an automatic gain control target of 8,000 and a maximum injection time of 50 ms. To minimize repeated analysis of the same ions, dynamic exclusion was set to 25 s.

### Statistical analysis

Statistical analyses were carried out using the GraphPad Prism software (version 8.3). Sample size estimation was conducted using the G*Power software (version 3.1). Data are presented as mean ± standard error of the mean. Differences were considered statistically significant at *P < 0.05, **P < 0.01, or ***P < 0.001, while *P* > 0.05 was regarded as not significant (NS). The Shapiro–Wilk test was used to evaluate the normality of data distributions before conducting comparisons between 2 groups. For experiments involving multiple groups, one-way analysis of variance (ANOVA) was used, and 2-way ANOVA was performed when 2 independent variables were analyzed. The number of biological replicates for each experiment was determined based on prior experience in our laboratory and information reported in related literature. In in vivo experiments, each “*n*” corresponds to an individual animal or specimen; in in vitro studies, each “*n*” represents an independent experimental sample.

## Data Availability

Data supporting the findings of this study can be obtained from the corresponding authors upon justified request.
