# Supplementary material for: Na+/K+-ATPase Modulates Purinergic P2X3 Receptor Function to Drive Bone Cancer Pain
Source: Research (Wash D C). 2025 Oct 20;8:0932. doi: 10.34133/research.0932 (PMC12536475; doi:10.34133/research.0932)
Supplement: Supplementary 1 — Figs. S1 to S10 Table S1 Data S1 and S2 [file research.0932.f1.zip › Supporting Information-Revised.docx]

**Supporting Information**

**Na^+^/K^+^-ATPase Modulates Purinergic P2X3 Receptor Function to Drive Bone Cancer Pain**

Songqiang Huang, Bo Peng, Wanting Dong, Jiapeng He, Hanbin Chen, and Jin-Song Bian

**Supporting figures and figure legends (Figs. S1–S4)**

**Supporting information for western blots (Figs. S5–S10)**

**Supporting table (Table S1)**

**Other supporting materials (Data S1, Data S2)**

**Supporting figures and figure legends**


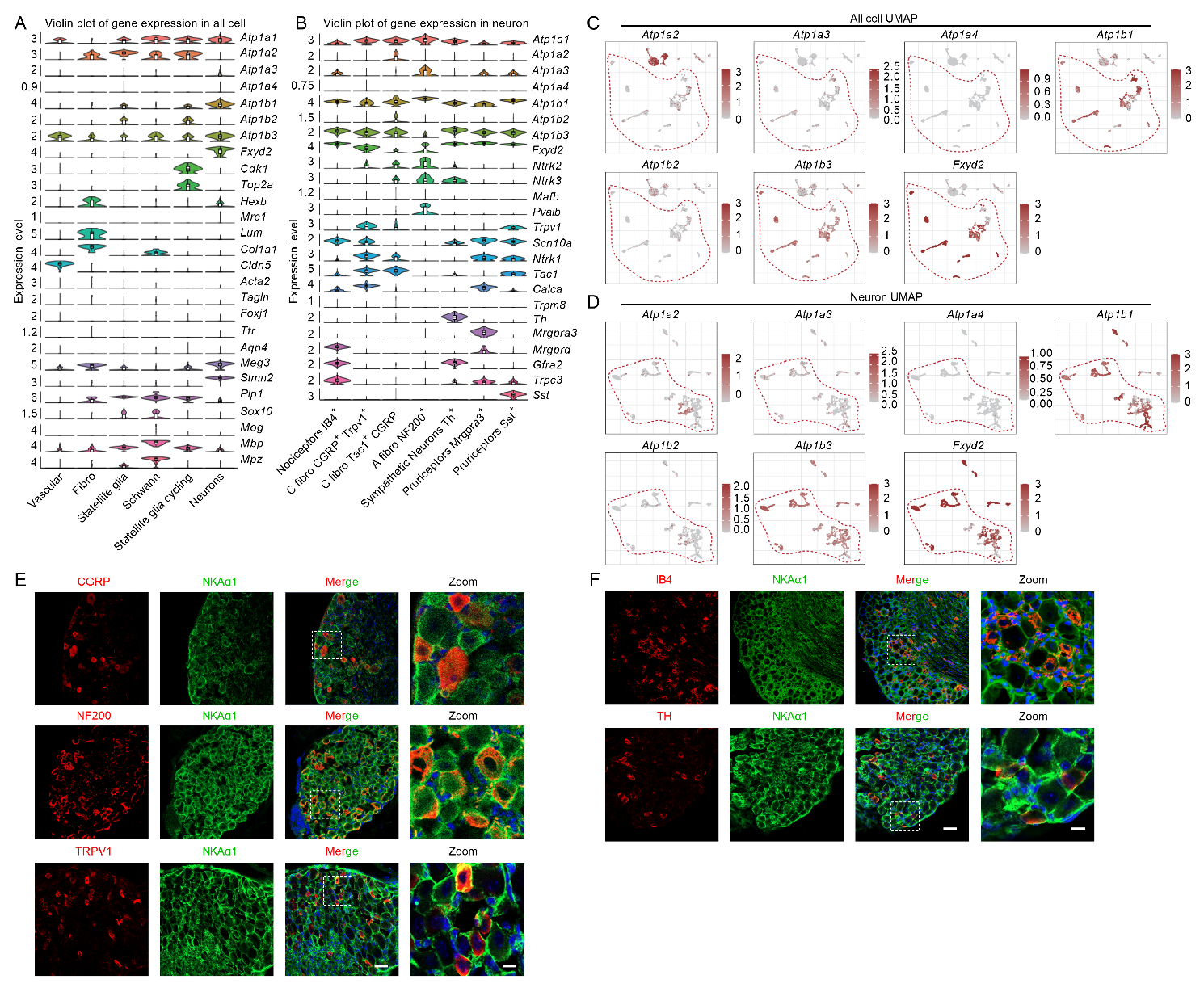


**Fig. S1.** Distribution of NKAα1 in various types of neurons in dorsal root ganglion (DRG). (A, B) Single-cell RNA sequencing (scRNA-seq) analysis showing the expression profiles of NKA subunits, including NKAα1, across various cell types (A) and specifically in neurons (B) within the DRG. (C, D) UMAP plots illustrating the distribution of NKA subunits, excluding NKAα1, in the DRG based on scRNA-seq analysis. (E, F) Representative images showing the colocalization of NKAα1 (green) and calcitonin gene-related peptide (CGRP, peptidergic C-fibers marker, red), neurofilament-200 (NF200, myelinated A-fibers marker, red), transient receptor potential vanilloid 1 (TRPV1, nociceptive neurons marker, red), isolectin B4 (IB4, non-peptidergic C-fibers marker, red) and tyrosine hydroxylase (TH, low-threshold C-fibers marker, red) in DRG. Scale bars in (E, F) indicate 50 [μm](https://baike.baidu.com/item/%CE%BCm/4897741) (left) and 10 [μm](https://baike.baidu.com/item/%CE%BCm/4897741) (right, Zoom).

**
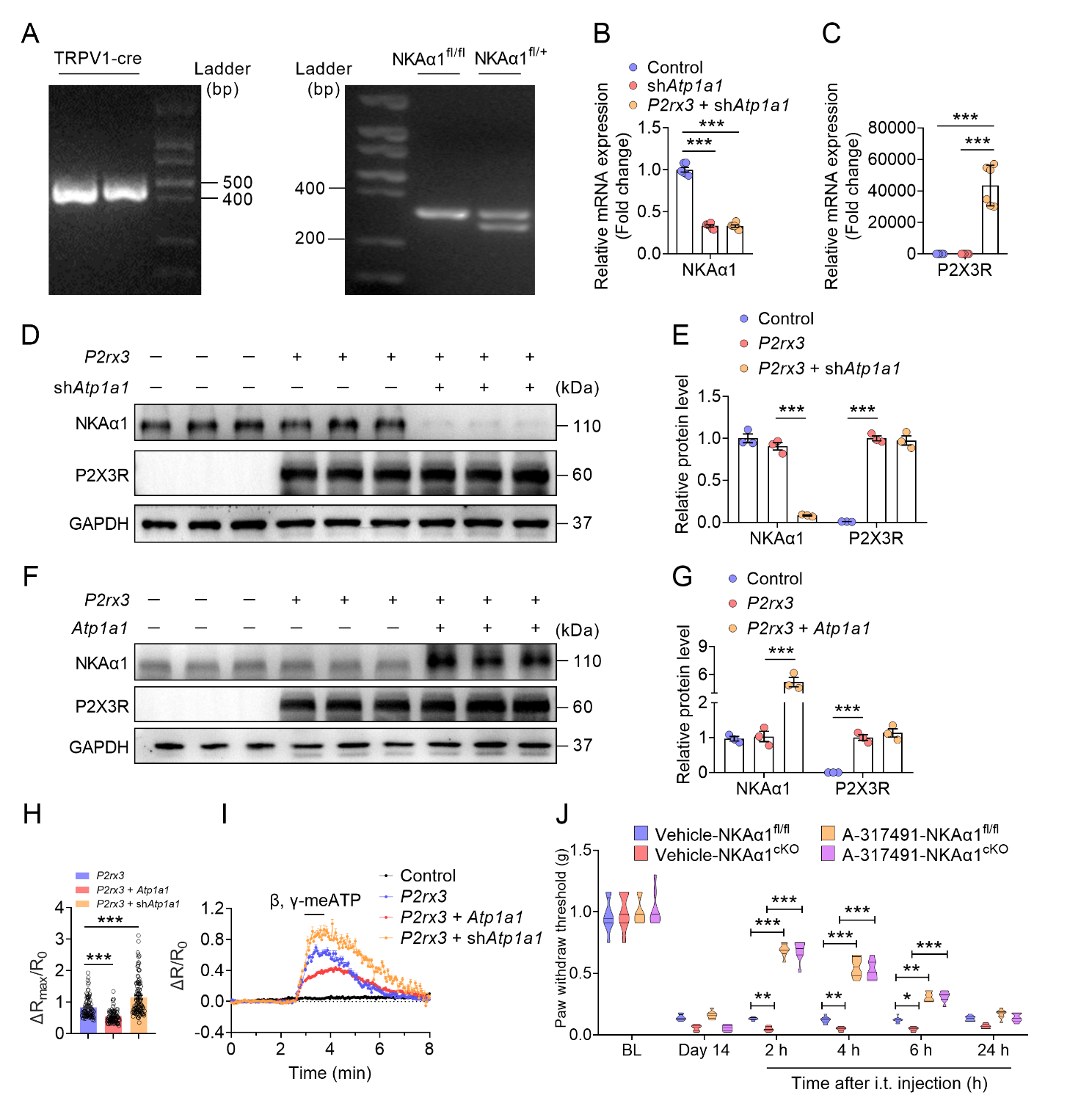
**

**Fig. S2.** Effects of NKAα1 knockdown and overexpression on the P2X3R-dependent Ca^2+^ influx. (A) Genotyping of Trpv1-cre, NKAα1^fl/fl^ and NKAα1^fl/+^ mice by polymerase chain reaction. NKAα1^fl/fl^ and NKAα1^fl/+^ represent homozygous and heterozygous mice, respectively. (B, C) Quantitative polymerase chain reaction (qPCR) analysis showing the mRNA expression of NKAα1 (B) and P2X3R (C) in HEK293T cells after NKAα1 silencing plasmid (sh*Atp1a1*) or P2X3R overexpression plasmid (*P2rx3*) treatment (n = 6). (D, E) Representative western blots (D) and analyses (E) of NKAα1 and P2X3R in HEK293T cells of control, *P2rx3* and *P2rx3 +* sh*Atp1a1* groups (n = 3). (F, G) Representative western blots (F) and analyses (G) of NKAα1 and P2X3R in HEK293T cells of control, *P2rx3* and *P2rx3 +* NKAα1 overexpression plasmid (*Atp1a1*) groups (n = 3). (H, I) Group data showing Ca^2+^ responses, and traces of time-dependent intracellular Ca^2+^ influx induced by β, γ-meATP (100 μM) treatment. (J) von Frey test showing the time course of mechanical allodynia after single intrathecal (i.t.) injection of A-317491 (10 mg/kg in saline) or vehicle in NKAα1^fl/fl^ and NKAα1^cKO^ mice after TCI treatment for 14 days (n = 8). Data are presented as mean ± SEM. Statistical analysis by one-way ANOVA with Bonferroni’s post hoc test in (B, C, E, G, H) or two-way ANOVA with Bonferroni’s post hoc test in (J). ^*^*p* < 0.05, ^**^*p* < 0.01, ^***^*p* < 0.001.


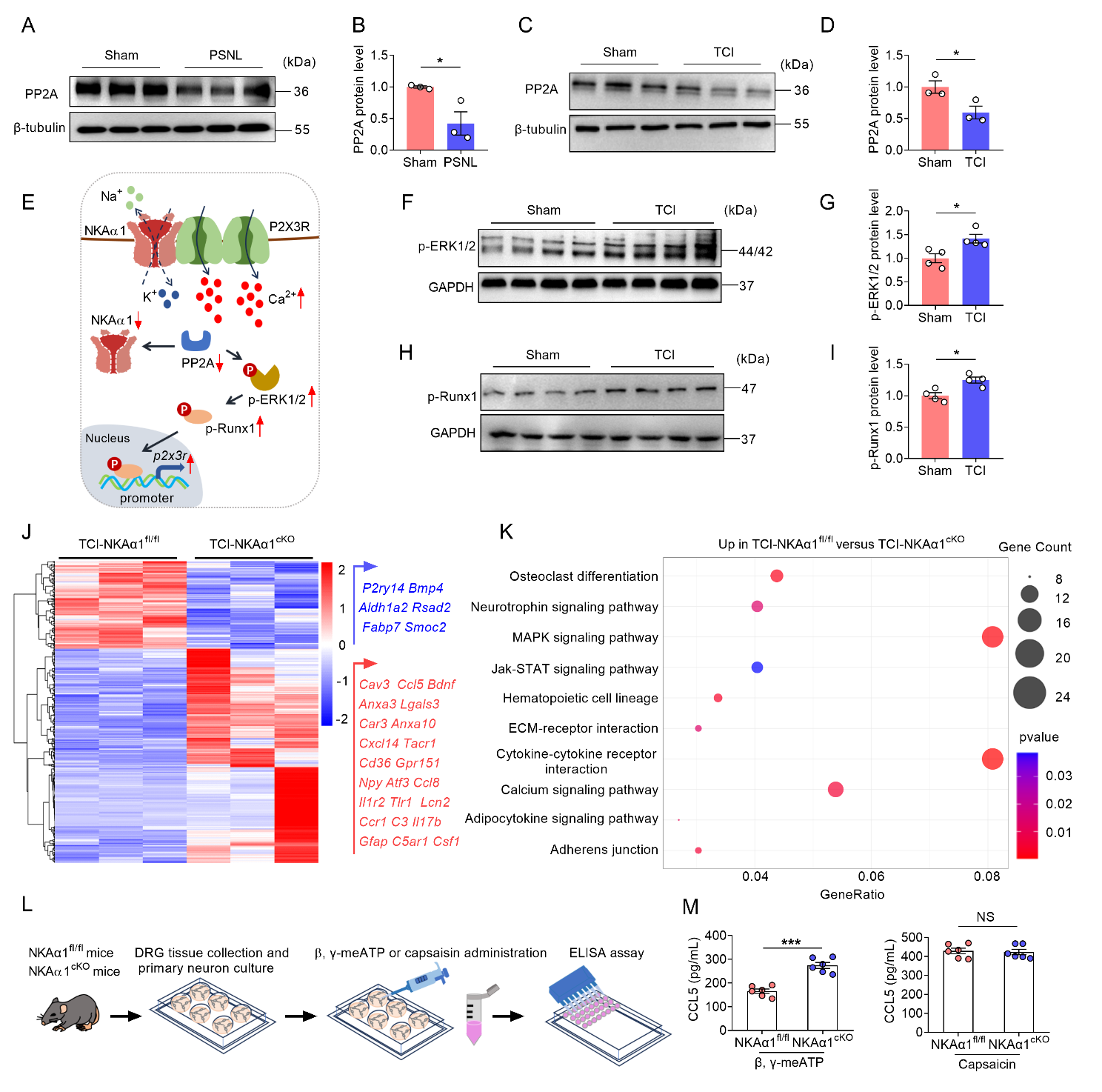


**Fig. S3.** Effects of tumor cell implantation (TCI) and partial sciatic nerve ligation (PSNL) treatment on protein expression levels. (A, B) Representative western blots (A) and analyses (B) of protein phosphatase 2A (PP2A) in dorsal root ganglion (DRG) of sham and PSNL groups (n = 3). (C, D) Representative western blots (C) and analyses (D) of PP2A in DRG of sham and TCI groups (n = 3). (E) The schematic diagram illustrates that reduced PP2A expression decreases NKAα1 membrane localization and activates ERK1/2-Runx1 signaling, ultimately enhancing *P2rx3* expression. (F-I) Representative western blots (F, H) and analyses (G, I) of p-ERK1/2 or p-Runx1 in DRG of sham and TCI groups (n = 4). (J) Heatmap of differentially expressed genes (DEGs) determined by whole-transcriptome RNA sequencing of DRG tissues from 3-month-old TCI-NKAα1^fl/fl^ and TCI-NKAα1^cKO^ mice. Normalized expression values (high, red; low, blue) were calculated for each DEGs (row). (K) Bubble charts enrichment analysis of upregulated genes in DRG tissues from TCI-NKAα1^fl/fl^ versus TCI-NKAα1^cKO^ mice. (L) Schematic of the experimental design. DRG tissues were isolated from NKAα1^fl/fl^ and NKAα1^cKO^ mice and used for primary neuronal culture. Cultured DRG neurons were subsequently stimulated with 100 μM β, γ-meATP or 1 μM capsaicin for 90 minutes. After treatment, culture supernatants were collected and subjected to ELISA analysis. (M) ELISA was performed to measure CCL5 levels in the supernatants of DRG neurons derived from NKAα1^fl/fl^ and NKAα1^cKO^ mice after treatment with β, γ-meATP or capsaicin (n = 6). Data are presented as mean ± SEM. Statistical analysis by unpaired Student’s t test in (B, D, G, I, M). NS: not significant. ^*^*p* < 0.05, ^***^*p* < 0.001.

**
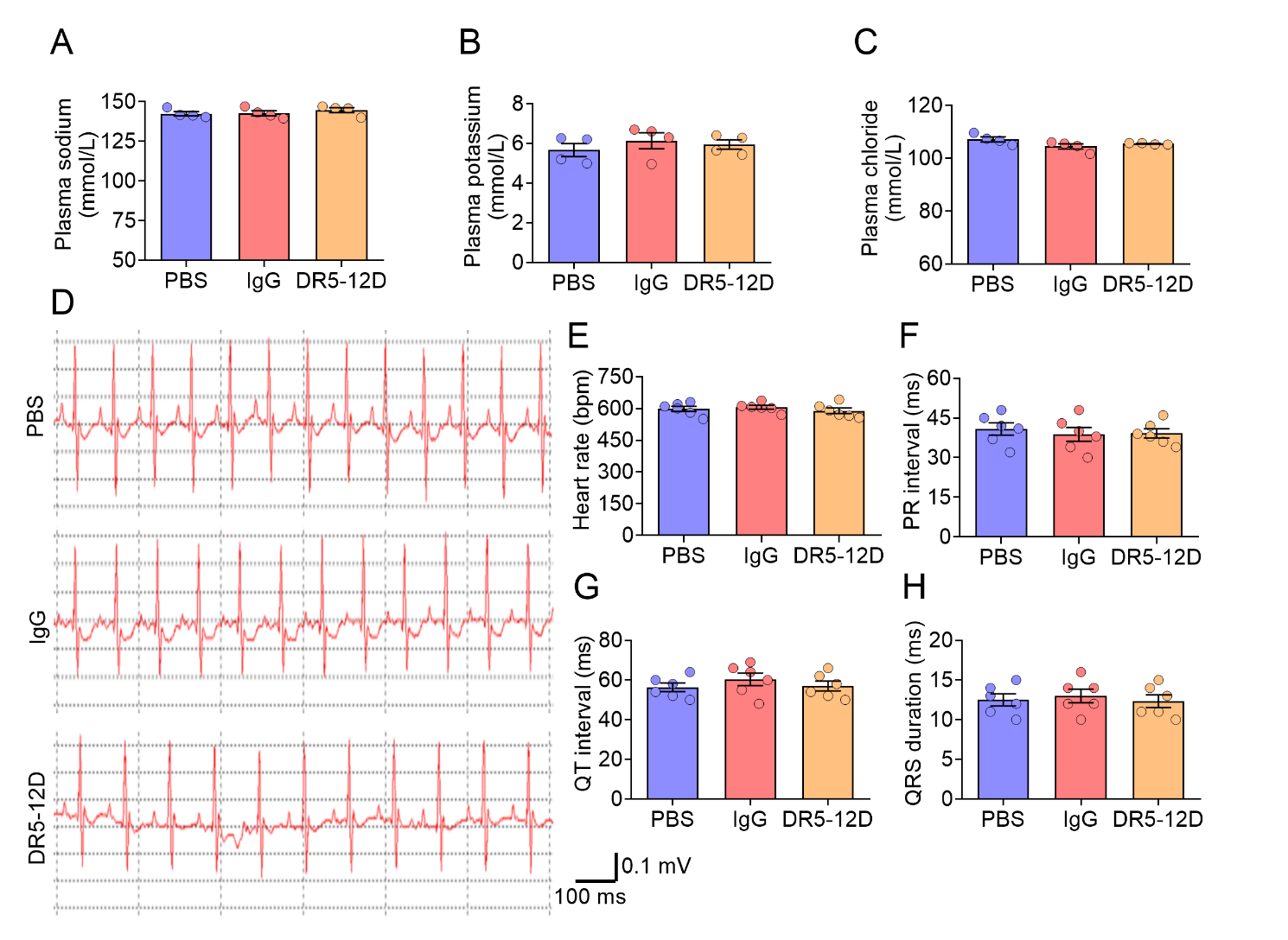
**

**Fig. S4.** Electrocardiographic recordings and serum electrolyte analyses following PBS, IgG, or DR5-12D treatment. (A–C) Quantification of plasma sodium (A), potassium (B), and chloride (C) levels in freshly collected plasma from mice treated with PBS, IgG, or DR5-12D for 7 consecutive days (n = 4). (D) Representative electrocardiographic traces from mice treated with PBS, IgG, or DR5-12D for 7 consecutive days. (E–H) Quantification of heart rate (E), PR interval (F), QT interval (G) and QRS duration (H) in the PBS, IgG, or DR5-12D groups (n = 6). Data are presented as mean ± SEM. Statistical analysis by one-way ANOVA with Bonferroni’s post hoc test in (A-C, E-H).

**Supporting Information for Western blots**

**
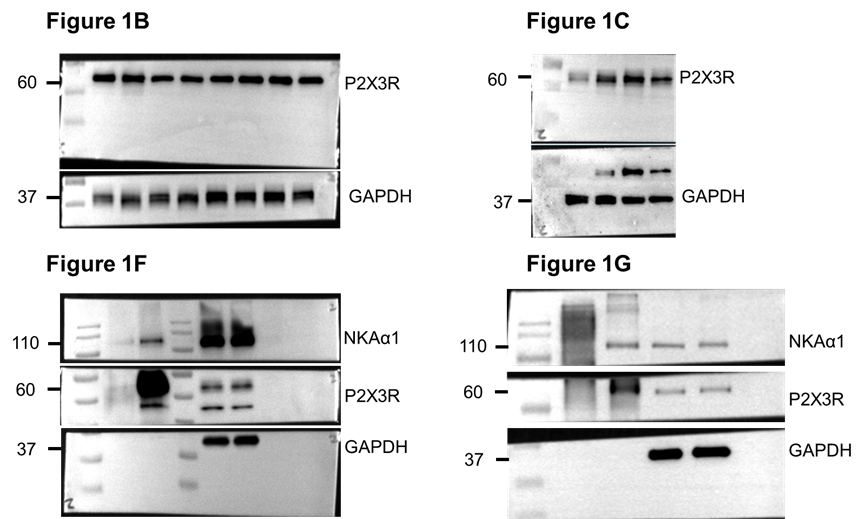
**

**Fig. S5.** Full original images of Western blotting assays for Figure 1.


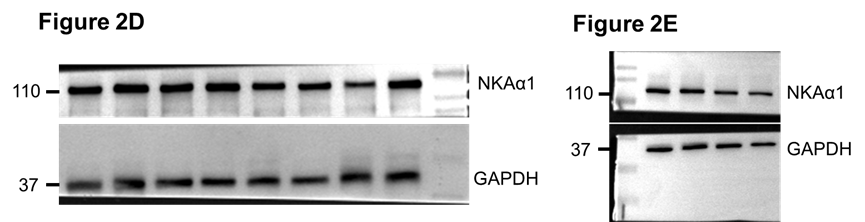


**Fig. S6.** Full original images of Western blotting assays for Figure 2.


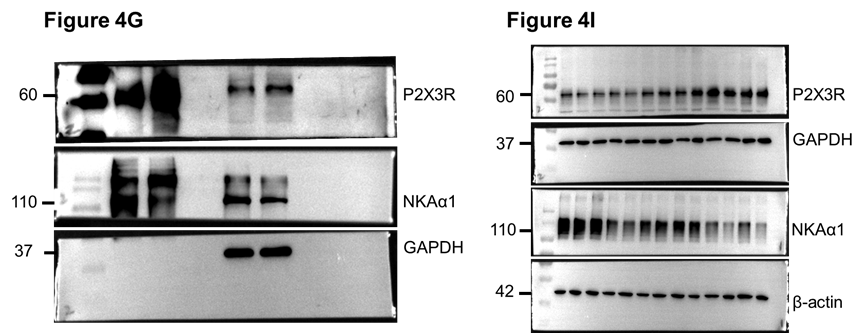


**Fig. S7.** Full original images of Western blotting assays for Figure 4.


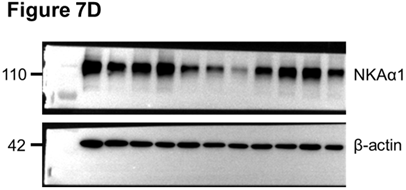


**Fig. S8.** Full original images of Western blotting assays for Figure 7.


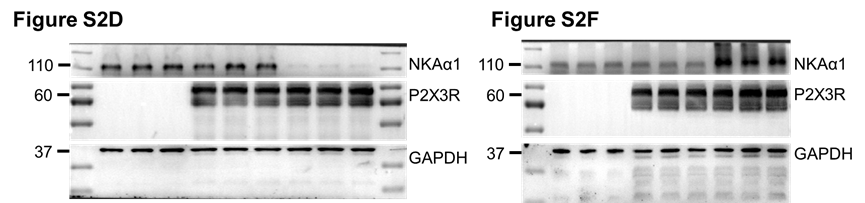


**Fig. S9.** Full original images of Western blotting assays for Figure S2.

**
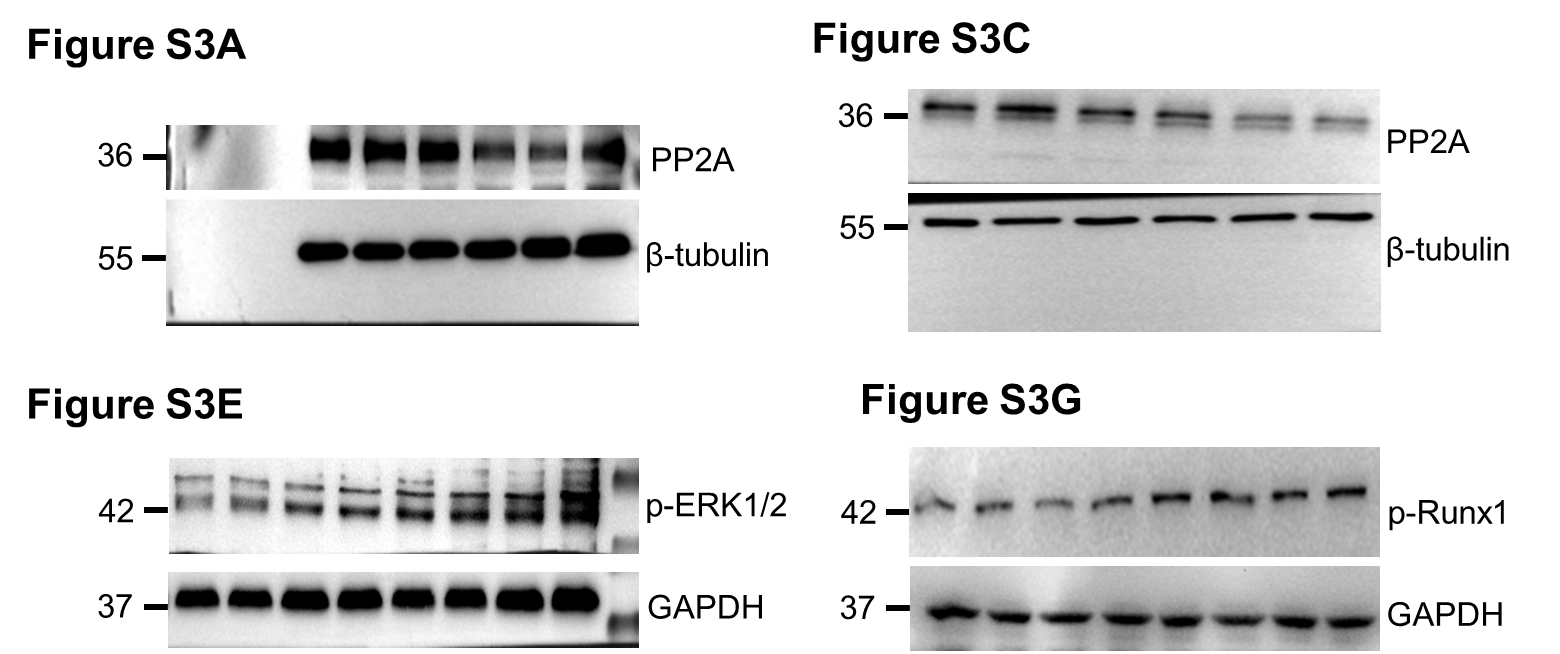
**

**Fig. S10.** Full original images of Western blotting assays for Figure S3.

**Supporting tables**

**Table S1.** Oligonucleotide primers for RT-qPCR.

| Names | Sequences (5’ to 3’) |
| --- | --- |
| ***Quantitative RT-PCR*** | |
| *P2rx1*-F | CGGATGGTGCTGGTACGAAA |
| *P2rx1*-R | CACTGACACACTGCTGATAAGG |
| *P2rx2*-F | GCGTTCTGGGACTACGAGAC |
| *P2rx2*-R | CGTACCACACGAAGTAAAGCA |
| *P2rx3*-F | AAAGCTGGACCATTGGGATCA |
| *P2rx3*-R | CGTGTCCCGCACTTGGTAG |
| *P2rx4*-F | ACCAGGAAACGGACTCTGTG |
| *P2rx4*-R | TCACGGTGACGATCATGTTGG |
| *P2rx5*-F | TGGAAGGGGTTCGTGTTGTC |
| *P2rx5*-R | AGGGAAGTGTCAATGTCCTGA |
| *P2rx6*-F | GGGGTTTCTGGATTACAAGACG |
| *P2rx6*-R | CCTATCACGTAGACTACCACTGC |
| *P2rx7*-F | GCACCGTCAAGTGGGTCTT |
| *P2rx7*-R | CAGGCTCTTTCCGCTGGTA |
| *Atp1a1*-F | GATCAGCATGGCCTATGGACAG |
| *Atp1a1*-R | ACCGTTCTCAGCCAGAATCACA |
| *Gapdh*-F | AACGACCCCTTCATTGAC |
| *Gapdh*-R | TCCACGACATACTCAGCAC |

Other supporting materials

Data S1. The list of 122 proteins (fold change > 2) identified by liquid chromatography tandem mass spectrometry in dorsal root ganglia of mice that interact with P2X3R.

Data S2. The list of 568 differentially expressed genes (P < 0.05 and fold change > 1.5) in dorsal root ganglia of TCI-NKAα1^fl/fl^ mice relative to TCI-NKAα1^cKO^ mice.
